# The Preventive Impact of Chokeberry (*Aronia melanocarpa* L.) Extract Regarding the Disruption of Calcium and Phosphorus Homeostasis and Chosen Pathways of Its Regulation in an Animal Model of General Population Exposure to Cadmium

**DOI:** 10.3390/nu17040702

**Published:** 2025-02-16

**Authors:** Małgorzata M. Brzóska, Małgorzata Gałażyn-Sidorczuk, Joanna Rogalska

**Affiliations:** Department of Toxicology, Medical University of Bialystok, Adama Mickiewicza 2C Street, 15-222 Bialystok, Poland; malgorzata.galazyn-sidorczuk@umb.edu.pl (M.G.-S.); joanna.rogalska@umb.edu.pl (J.R.)

**Keywords:** *Aronia melanocarpa* L. berry extract, cadmium, calciotropic hormones, calcitonin, calcium, chokeberry, Klotho, parathormone, inorganic phosphorus, vitamin D_3_ metabolites

## Abstract

**Background:** Our previous research in an experimental model of current environmental human exposure to cadmium (Cd) (female rats fed a diet containing Cd at 1 and 5 mg/kg for up to 2 years) revealed that chronic treatment with this toxic element destroyed the metabolism of the bone tissue, decreased mineralisation, and weakened bone biomechanical properties, whereas the co-administration of a 0.1% chokeberry (*Aronia melanocarpa* L. (Michx.) Elliott berry) extract (AME) ameliorated the osteotoxic action of Cd. **Methods:** In this study, it was explored whether the unfavourable effect of Cd and the protective action of AME might be mediated by the impact on the metabolism of bone essential elements such as calcium (Ca) and inorganic phosphorus (P_i_), including the pathways of its regulation by calciotropic hormones (parathormone—PTH, calcitonin—CT, and 1,25-dihydroxyvitamin D_3_—1,25(OH)_2_D_3_) and Klotho. **Results:** Low-level Cd treatment (1 mg/kg) caused only a temporary elevation in the serum PTH concentration and a decline in the concentration of CT. Moderate treatment with Cd (5 mg/kg) destroyed the body homeostasis of both mineral elements (lowered their concentrations in the serum and enhanced urinary loss), influenced the serum concentrations of Klotho and calciotropic hormones, as well as reduced the concentrations of 25-hydroxyvitamin D 1alpha-hydroxylase (1alpha-OHase) and 1,25(OH)_2_D_3_ in the kidney. The application of AME during Cd intoxication improved the pathways involved in maintaining Ca and P_i_ homeostasis and allowed subjects to maintain the proper levels of these elements in the serum and urine. **Conclusions:** In conclusion, Cd at low-to-moderate exposure may exert an unfavourable impact on bone by influencing the pathways involved in regulating Ca and P_i_ metabolism and destroying the body status of these minerals. It seems that the possible mechanism of the osteoprotective effect of AME during chronic intoxication with this toxic element involves normalization of the concentrations of calciotropic hormones and Klotho in the serum and improvement of the homeostasis of Ca and P_i_. This study provided further evidence that chokeberry products may be an effective strategy in counteracting the unfavourable effects of chronic low-to-moderate exposure to Cd.

## 1. Introduction

Bone diseases, including osteoporosis and bone fractures, are civilization diseases, the prevalence of which is increasing worldwide [1,2,3]. It is well known that an important role in the pathogenesis of these diseases has been played by nutritional deficiencies, including, first of all, bone essential macro- and micronutrients such as calcium (Ca), inorganic phosphorus (P_i_), and vitamin D_3_, as well as by numerous other factors that disturb mineral metabolism [2,3]. Recently, due to increased chemical exposure in daily life, common contamination of the environment and food, and thus human exposure to numerous chemical substances during a lifespan, there is growing interest in xenobiotics, including cadmium (Cd), as potential risk factors for bone diseases [4,5,6,7,8].

**Figure 1:**
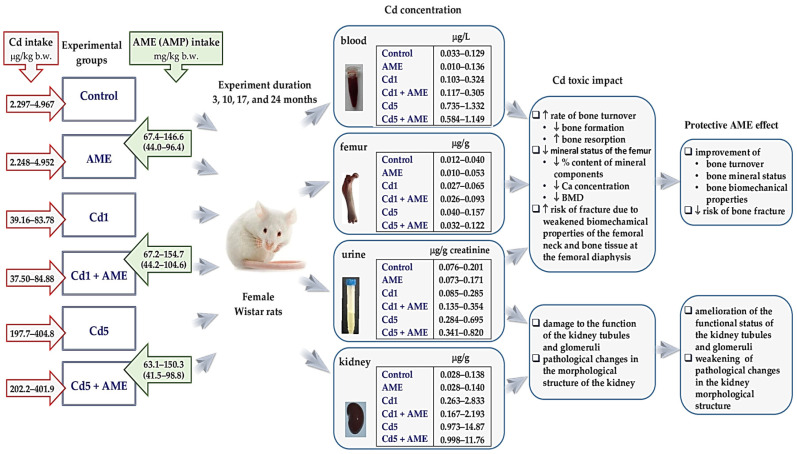
Diagram of our experimental animal model created to investigate the impact of a lifetime human exposure to cadmium (Cd) and the protective effect of *Aronia melanocarpa* L. berry extract (AME) [17], as well as the main findings of the study concerning the bone (Table S1) [18,19] and kidney (Table S2) [21]. Female rats were fed with commercial rodent fodder with added Cd at 0, 1, or 5 mg/kg (the control group, Cd1 group, and Cd5 group, respectively) and/or were supplemented with a 0.1% aqueous AME (the AME group, Cd1 + AME group, and Cd5 + AME group). The experiment lasted up to 24 months to cover all stages of the animal’s life and to reflect the age of about 60–70 years in people [38,39]. Thus, this study started with post-weaning rats and was conducted through growth into adulthood and the phase of adulthood up to the elderly. To assess the effects of Cd exposure and AME supplementation in different stages of life (youth, adulthood, and the elderly), the measurements were made after 3, 10, 17, and 24 months. The concentration of Cd in the rodents’ blood, urine, kidney, and bone tissue fed a diet with this toxic element added at 1 and 5 mg/kg [17] ranged within the values currently determined among the worldwide population [4,8,11,12,13]. The administration of 0.1% AME was a source of higher (2.9–7.3 times) 24 h intake of polyphenolic compounds (AMP) than the average consumption of these compounds in humans (1000 mg [40]; 14.29 mg/kg body weight—b.w. [21]). The arrows in the figure indicate the directions of changes (↑, increased; ↓, decreased). The photos presented in this diagram come from the authors’ collections.

Environmental exposure to Cd in developed and developing countries is inevitable nowadays [9,10,11,12]. Although the current lifetime exposure to this xenobiotic is generally low to moderate [4,8,11,12,13], it creates an important problem for public health [9,10,11,12]. Epidemiological studies provide more evidence that this exposure may contribute to the development of numerous diseases, including osteoporosis with bone fractures, kidney and liver diseases, cardiovascular diseases, neurodegenerative disorders, diabetes, and cancers [4,5,6,8,9,11,13,14,15,16]. Our study, performed in an experimental model of lifetime general population intoxication with this trace element (female rats fed a diet containing Cd at 1 and 5 mg/kg for up to 2 years, which corresponds to low-level and moderate environmental exposure, respectively), also disclosed the unfavourable effect of even relatively low long-lasting exposure to Cd on various organs and systems, including the skeletal system and kidney, which is an organ whose functioning has a significant impact on bone tissue metabolism (Figure 1, Tables S1 and S2) [17,18,19,20,21,22,23].

For most people, the inevitable environmental exposure to Cd during a lifespan and the available data from epidemiological studies on the harmful effects on human health of even low-level intoxication [4,5,8,9,11,12,13,14,15,16] justify the need to recognise the risk of health damage at current levels of exposure and to develop an effective strategy to counteract or at least minimise the impact. Considering these, scientists, including nutritionists, have drawn attention to the possibility of using nutritional factors as an effective protective strategy against Cd gastrointestinal absorption and the outcomes of its action [7,10,24,25,26,27,28]. Data from the literature indicate that the promising strategy for alleviating Cd toxicity may be plant remedies rich in certain bioactive substances, mainly including polyphenols, carotenoids, vitamins, and essential elements [7,10,24,25,26,27,28]. The results of our widely designed research suggest that chokeberry-based products (*Aronia melanocarpa* L. berries (Michx.) Elliott, Rosaceae) seem to be this strategy [17,18,19,20,21,22,23].

Both epidemiological [4,5,6,8] and experimental [5,7,18,19,20,29,30] data show that the kidneys and bones are at the highest risk of damage under chronic Cd intoxication. It is known that this toxic heavy metal damages the bone directly by affecting osteoblast and osteoclast activity, as well as indirectly by destroying the mineral metabolism, mainly the homeostasis of Ca and phosphates as a result of their lowered absorption from the digestive tract, elevated loss in the urine (due to kidney damage), and the destroyed metabolism of calciotropic hormones [5,18,20,29,30,31]. Ca and P_i_ (in the form of phosphates) are the main mineral components of bone [30,32]; however, the role of these elements is not limited to building the skeleton. Ca regulates numerous physiological processes, including the function of enzymatic and non-enzymatic proteins, cellular signalling, muscle contractility, and blood coagulation [33]. Like Ca, phosphates also regulate the function of various proteins, including enzymes, and cellular signalling [34]. Because maintaining Ca and P_i_ homeostasis is crucial for metabolic processes and organ functioning, recognising the influence of Cd on the homeostasis of these minerals in the body is important, not only for explaining its osteotoxicity.

Our recent experiment, performed in a rodent model (female rats) of human exposure to environmental Cd, has revealed that even low exposure (Cd in a diet at the concentration of 1 mg/kg) destroyed bone metabolism, leading to inappropriate bone mineral status (decreased amount of minerals, including Ca, and lower bone mineral density—BMD) and weakened biomechanical properties of bone, indicating enhanced risk of fractures (Figure 1, Table S1) [18,19,20]. These studies showed that the mechanism of osteotoxic Cd action at low-level and moderate treatment involves the stimulation of bone resorption and inhibition of bone formation, and it is related to destroying the bone tissue oxidative/antioxidative balance [18,19,20]. Since, in our study, even low Cd intoxication (1 mg/kg of diet) led to kidney damage (Figure 1, Table S2) [21], it seems possible that its action on the bone was also mediated by the damaging impact on the kidney, whose proper functioning is important for the metabolism of minerals and vitamin D_3_ [35]. Cd has been reported to disrupt the hormonal regulation of the balance of Ca and P_i_ in both humans [36,37] and experimental animals [30,31], but the risk of disordering skeletal metabolism at low chronic Cd intoxication and the mechanisms of the impact, including the effect on the metabolism of these minerals and the pathways of its regulation, are unknown. Although at the current stage of our research, not all mechanisms of osteotoxicity of this trace element at low-level and moderate exposure have already been explained [18,19,20], it is important to emphasise that the disrupting influence of this xenobiotic on the metabolism, the biomechanical properties of the skeleton, and the morphological structure and functional status of the kidney was counteracted by concomitant supplementation with a 0.1% aqueous extract from *A. melanocarpa* berries (AME) (Figure 1, Tables S1 and S2) [18,19,20]. These findings show the possibility of effective protection against the damaging influence of Cd on the critical organs of this xenobiotic under chronic intoxication, such as on the bones and kidneys.

Considering the above, we have formulated a hypothesis that the unfavourable Cd action on the bone and the protective impact of AME might be related to influencing the homeostasis of Ca and P_i_ via disrupting the mechanisms of its regulation, especially hormonal regulation, in which calciotropic hormones, such as parathormone (PTH), calcitonin (CT), and an active metabolite of vitamin D_3_—1,25-dihydroxyvitamin D_3_ (1,25(OH)_2_D_3_)—are involved [37,41]. The current investigation aimed to resolve this hypothesis. Thus, the effects of Cd and AME administered alone and in conjunction on the metabolism of the main bone minerals, i.e., Ca and P_i_, and the pathways of its regulation were investigated in our experimental model of human exposure to this trace element (Figure 1) [17,18,19,20,21,22,23]. The serum concentrations of Ca and P_i_ (the main markers of their homeostasis in the organism) and renal handling of these elements were estimated. Addtionally, the concentrations of 1,25(OH)_2_D_3_, CT, and PTH in the serum and 25-hydroxyvitamin D 1alpha-hydroxylase (1alpha-OHase) and 1,25(OH)_2_D_3_ in the kidney were determined. To evaluate the renal handling of Ca and P_i_, their total 24 h excretion in urine and fractional excretion (percentage of the element filtered at the renal glomeruli that does not undergo reabsorption into the tubular fluid) were evaluated. The serum concentration of Klotho, as a protein regulating Ca and P_i_ homeostasis [42,43,44], was also assayed. Moreover, to assess the relationship between Cd’s impact on the metabolism of Ca and P_i_ and its hormonal regulation and this toxic heavy metal’s influence on bone metabolism and mineral density, as well as the possible role of AME in the mechanisms of its protective influence against the osteotoxic action of Cd, mutual dependencies between the indices of these essential elements’ metabolism, markers of the pathways of their regulation, and previously reported main markers of bone status [18,19] and kidney function in these animals [21] were evaluated. The relationships between the indices of the metabolism of Ca and P_i_ and the pathways of its regulation and already reported concentration of Cd in the blood, urine, bone, and kidney [17] were also estimated.

## 2. Materials and Methods

### 2.1. Laboratory Animals and Ethical Rules in Animal Research

The Local Ethics Committee for Animal Experiments in Bialystok (Poland) approved the experimental protocol using laboratory animals (approval No. 60/2009 on 21 September 2009). All experimental animal use procedures complied with the ethical principles and institutional guidelines and the International Guide for the Use of Animals in Biomedical Research.

A total of 192 young (3–4 weeks old) female Wistar rats (Hannover Wistar rats bred using the Charles River International Genetic Standardization Program—Crl: WI (Han)) provided by the certified laboratory animal breeding facility in Brwinów (Poland) were used. This number of animals allowed for a reliable statistical analysis of the results and their unambiguous interpretation. In addition, due to the long duration of the experiment (2 years), the fact that rats could die of natural causes was taken into account.

Because the investigation was designed to estimate the impact of low-level treatment with Cd, females were the more appropriate gender than males due to their higher susceptibility to this heavy metal’s toxicity. In experiments conducted on rats and epidemiological studies in humans, it has been revealed that females have higher Cd concentrations in the blood and urine and are more vulnerable to this xenobiotic-induced damage to some organs and systems, including the kidney and skeleton [4,8,12,16,30,45,46]. The sex-related differences in the metabolism and toxicity of Cd are caused by biological factors such as menstruation, pregnancy, lactation, and menopause in women [45,46]. Lower iron (Fe) stores in women’s bodies result in higher gastrointestinal absorption of Cd [45,46]. Moreover, Ca deficiency (due to pregnancy and lactation) and hormonal changes after menopause make females especially susceptible to the unfavourable impact of this heavy metal on bone [4,8,10,45]. The use of female rats is in line with the current principles regarding animal testing, particularly the 3Rs (Replacement, Reduction, Refinement), which recommend reducing the number of animals and choosing a sex that is more vulnerable to a tested substance.

The newly arrived animals were allowed a 5-day adaptation to experimental conditions before being used in the study. Since all females were healthy, they were included in the study. During acclimation and throughout the study, the rats were kept in cages (made of stainless steel), 4 individuals in each, under an automatically controlled environment (12 h of light and 12 h of darkness schedule, relative air humidity 50 ± 10%, and temperature of 22 ± 2 °C). During the experiment, the location of the rodent cages in the animal house was changed to avoid the confounding influence of the cage location. The animals were provided with free access to drinking water and feed.

### 2.2. Labofeed Diets with Added Cd

Since diet is the main source of exposure of the general population to Cd [9,10], in our experimental model, to reflect current human environmental exposure to this toxic element, the rodents were treated with this element in a diet. Cd-containing diets were prepared (Label Food “Morawski’’; Kcynia, Poland) by adding cadmium chloride (CdCl_2_ × 2.5 H_2_O; POCh, Gliwice, Poland) to the components of the standard breeding diet (Labofeed H diet used from the beginning of the study up to the end of its 3rd month) and maintenance diet (Labofeed B diet administered since the start of the 4th month) at the production stage to obtain concentrations of this element reaching 1 and 5 mg/kg. The quantification of Cd revealed that its concentration (mean ± standard deviation—SD) in the feed (1.09 ± 0.13 mg/kg was determined in the 1 mg/kg diet and 4.92 ± 0.53 mg/kg—in the 5 mg/kg diet) reached the certified values [17]. The standard Labofeed diets with no addition of CdCl_2_ × 2.5 H_2_O contained Cd at the average concentration of 0.0584 ± 0.0049 mg/kg [17].

The Labofeed H diet contained 1.1% Ca, 0.8% P, and vitamin D_3_ in the amount of 2000 j.m./kg. The content of these ingredients in the Labofeed B diet reached 1%, 0.75% P, and 1500 j.m./kg, respectively (manufacturer’s data).

### 2.3. A. melanocarpa Berry Extract

AME was administered as a 0.1% solution prepared daily from commercial lyophilised chokeberry extract (Adamed Consumer Healthcare; Tuszyn, Poland) by dissolving it in redistilled water. The powdered extract contained polyphenols (65.74%, including 18.65% of anthocyanins), phytosterols, carotenoids, pectins, triterpenes, sugar, sugar alcohols (sorbitol and parasorboside), minerals, and vitamins (manufacturer’s Certificate KJ 4/2010). Other components such as dietary fibre, tannins, carbohydrates, proteins, and organic acids (citric acid and L-malic acid) were also present in the extract [18,47].

The 0.1% AME contained polyphenols at the concentration of 612.40 ± 3.33 ng/mL (mean ± standard error—SE) and was stable for 24 h after preparation [18]. The ultra-performance liquid chromatography analysis of the polyphenolic profile (presented in [18] and at https://www.mdpi.com/article/10.3390/ijms241411647/s1 [21] (accessed on 14 November 2024)) disclosed that 1 mL of the extract contained 202.28 ± 1.28 ng of total anthocyanins (including 80.07 ± 1.05 ng of cyanidin 3-O-β-galactoside, 33.21 ± 0.01 ng of cyanidin 3-O-α-arabinoside, and 3.68 ± 0.01 ng of cyanidin 3-O-β-glucoside), 129.87 ± 1.12 ng of proanthocyanidins, 110.92 ± 0.89 ng of phenolic acids (including 68.32 ± 0.08 ng of chlorogenic acid), and 21.94 ± 0.98 ng of flavonoids [18]. The Cd concentration in the 0.1% AME was <0.05 ng/mL (limit of detection of the atomic absorption spectrometry—AAS method) [17].

### 2.4. Experimental Protocol

After acclimation to the laboratory conditions, the females were randomly allocated into six groups (32 individuals per group) and fed a diet without and with added Cd (0, 1, or 5 mg/kg) and redistilled water or 0.1% aqueous AME according to the diagram shown in Figure 1 (the control, AME, Cd1, Cd1 + AME, Cd5, and Cd5 + AME groups). The rats had unrestricted access to drinking fluids—redistilled water free of contaminants (the control, Cd1, and Cd5 groups) or 0.1% AME (the AME, Cd1 + AME, and Cd5 + AME groups) and rat chow (Labofeed H diet for the first 3 months and Labofeed B diet in the following months) with Cd (the Cd1, Cd1 + AME, Cd5, and Cd5 + AME groups) or without added this heavy metal (the control group and AME group). The AME, Cd1 + AME, and Cd5 + AME groups received only 0.1% AME to drink.

Animals’ clinical observations throughout the study revealed no signs of Cd toxicity. The daily consumption of drinking water and feed and the body weight gain in the animals given Cd throughout the experiment did not differ from the control group [17]. The females did not show any abnormalities in their behaviour or the appearance of their external coverings, body cavities, or mucous membranes, nor any symptoms that could indicate disease or suffering. However, spontaneous deaths of one female in the control group, Cd1 group, and Cd5 group were noticed between the 17th and 24th month of the experiment, and these three groups consisted of 7 females after 24 months [17]. The body weight and consumption of food and drinking fluids did not differ among the experimental groups [17]. There were no differences in the daily intakes of Cd (the Cd1 vs. the Cd1 + AME groups and the Cd5 vs. the Cd5 + AME groups) and AME (the AME, Cd1 + AME, and Cd5 + AME groups) (Figure 1) [17]. More details about the experimental model are given in previous articles [17,18,19,20,21,22,23].

After the 24 h collection of urine performed in metabolic cages after 3, 10, 17, and 24 months of the study [22], the females were given no food for the night, and then they underwent barbiturate (Morbital, 30 mg/kg b.w., intraperitoneally; Biowet, Pulawy, Poland) anaesthesia. Whole blood was taken via cardiac puncture without and with an anticoagulant (heparin; Biochemie GmbH, Kundl, Austria). Macroscopic examination during the necropsy showed no abnormalities in the internal appearance of the body cavities (abdominal and pelvic cavities, thoracic cavity, and cranial and neck cavities). Various organs were collected and evaluated macroscopically (no pathological changes were noted), including the kidneys used in the present study. After dissection, the kidneys were rinsed with ice-cold physiological saline (0.9% sodium chloride; POCh, Gliwice, Poland) and gently dried on filter paper. The kidneys were stored at −80 °C until they were used.

The experimental procedures did not involve any suffering of the animals. However, if any of the females showed signs of toxicity or any symptoms of suffering during the experiment, that individual would be killed immediately for humane reasons. The rats could only feel discomfort during their stay in metabolic cages with limited space (one animal in the cage) and short-term stress at the intraperitoneal injection of the anaesthetic.

### 2.5. Analytical Procedures

#### 2.5.1. Measurement of the Concentration of Ca in the Serum and Urine and Evaluation of Its Fractional Excretion in the Urine

The concentration of Ca was determined by the high-resolution AAS method with atomisation in an acetylene/nitrous oxide burner flame at the analytical wavelength for this element of λ = 422.6728 nm using a continuous-source ContrAA^®^ 700 atomic absorption spectrometer (Analytik Jena AG, Jena, Germany). Standard solutions of Ca and the serum and urine (24 h) samples were diluted using an aqueous solution containing 0.1% lanthanum chloride and 0.1% caesium chloride (both reagents from Merck KGaA, Darmstadt, Germany). The correctness of the procedures was checked and confirmed by determining this element concentration in certified reference materials (Table 1).

Based on the concentrations of Ca and creatinine in the urine and serum (creatinine was previously assayed [21]), the fractional urinary excretion of Ca (FE–Ca) was calculated. The following formula was used: FE–Ca = (creatinine concentration in the serum × Ca concentration in the urine × 100)/(Ca concentration in the serum × creatinine concentration in the urine). For these calculations, the concentrations of Ca and creatinine in the biological fluids were expressed as mg/mL.

#### 2.5.2. Measurement of the Concentration of P_i_ in the Serum and Urine and Evaluation of Its Fractional Excretion in the Urine

The concentration of P_i_ in the serum and 24 h urine was assayed using a kit by BioMaxima (No. 1-403-0200; Lublin, Poland) and Specord 50 Plus (Analityk Jena, Jena, Germany). The assay was based on the molybdate method (the reaction of coupling of phosphorus ion with molybdenum ion into the phosphomolybdenum complex, quantified spectrophotometrically at 340 nm). The analytical quality of the measurement of P_i_ was checked with the use of control serum BioNorm (No. 1-801-0020) by BioMaxima (Lublin, Poland). P_i_ concentration assayed in the certified serum (3.811 ± 0.020 mg/100 mL; mean ± SD) was consistent with the reference concentration (3.10–4.20 mg/100 mL). The measurement precision, expressed as CV, was <4.5% for the serum and <8% for the urine.

Based on the serum and urinary concentrations of P_i_ and creatinine [21], the fractional urinary excretion of P_i_ (FE–P_i_) was calculated from the following formula: FE–P_i_ = (creatinine concentration in the serum × P_i_ concentration in the urine × 100)/(P_i_ concentration in the serum × creatinine concentration in the urine). For these calculations, P_i_ and creatinine concentrations in the serum and urine were expressed as mg/mL.

#### 2.5.3. Quantification of Calciotropic Hormones in the Serum

1,25(OH)_2_D_3_, PTH, and CT were quantified in the serum using rat-specific kits based on a double-antibody sandwich enzyme-linked immunosorbent assay (ELISA) by SunRed (Shanghai, China). 1,25(OH)_2_D_3_ was determined with the use of a Rat (DVD/DHVD3) ELISA Kit (No. 201-11-0001). The intra-assay CV for two used kits was <4.8% and <4.9% and the inter-assay CV was <2%. PTH was measured with a Rat (i-PTH) ELISA Kit (No. 201-11-0516). The intra-assay CV was <3.5% for the first used kit and <4.8% for the second kit, whereas the inter-assay CV was <4.5%. CT was determined using a Rat (CT) ELISA Kit (No. 201-11-0347). The intra-assay CV for the used kits was <5% and <3%, respectively, whereas the inter-assay CV reached <5%.

The measurements of calciotropic hormones, like other assays performed using commercial kits, were carried out following the producers’ recommendations described in the instructions included in particular kits. The assays based on the ELISA method were conducted with an Epoch spectrophotometer (Bio Tek Instruments, Inc., Winooski, VT, USA).

#### 2.5.4. Determination of Klotho Concentration in the Serum

The concentration of Klotho was measured in the serum with a Rat Kl(Klotho) ELISA Kit (No. ER0658) by FineTest (Wuhan, China), based on sandwich ELISA. The intra-assay CV reached <6% and <3% for the used kits. The inter-assay CV was <7%.

#### 2.5.5. Determination of 1alpha-OHase and 1,25(OH)_2_D_3_ Concentrations in the Kidney

The concentrations of 1alpha-OHase and 1,25(OH)_2_D_3_ were determined in aliquots of 10% homogenates. The homogenates were prepared as described in our previous study [21]. Known weight slices of the right kidney (weighed with an accuracy of 0.0001 g using a RADWAG^®^ analytical balance from Radom, Poland) were subjected to homogenisation in a cold 50 mM potassium phosphate buffer pH = 7.4 (prepared from potassium dihydrogen phosphate and dipotassium hydrogen phosphate purchased from POCh, Gliwice, Poland) containing added butyl-hydroxytoluene (Sigma-Aldrich GmbH; Steinheim, Germany) in acetonitrile (Merck, Darmstadt, Germany) as described [21]. The homogenates were prepared using an Ultra-Turrax T25 high-performance homogeniser (IKA, Staufen, Germany) and immediately centrifuged (Medical Instruments MPW-350R centrifuge; Warsaw, Poland) at 10,000× *g* for 5 min at 4 °C [21].

The 1alpha-OHase concentration in the kidney homogenate aliquots was measured using a Rat Cyp27b1(25-hydroxyvitamin D-1 alpha hydroxylase, mitochondrial) ELISA Kit (No. ER0420) by FineTest (Wuhan, China). The intra-assay CV was <8% and <4% for two used kits, whereas the inter-assay CV was <8%. The 1,25(OH)_2_D_3_ concentration in the aliquots was measured as described for the measurements of this metabolite of vitamin D_3_ in the serum (Rat (DVD/DHVD3) ELISA Kit No. 201-11-0001 by SunRed). The intra-assay CV was <2% and <5% for the first and second used kits, respectively, and the inter-assay CV reached <6%.

### 2.6. Statistical Analysis

The Statistica 13.3 software package (StatSoft, Tulsa, OK, USA) was used for all statistical analyses. Initially, the normality of the distribution of particular tested variables in all experimental groups was checked by performing the Shapiro–Wilk test. Next, the Kruskal–Wallis test (the rationale was no normal data distribution) was conducted to verify the occurrence of statistically significant (*p* < 0.05) differences between experimental groups. The results for each group are presented in figures as the minimum and maximum values for 8 rats (only after 24 months were there 7 females in the AME, Cd1, and Cd5 groups), 25–75% confidence interval, and median values. In the figures, statistically significant differences compared to the control group, the appropriate group administered Cd alone (the Cd1 + AME group vs. the Cd1 and the Cd5 + AME group vs. the Cd5 group), and the appropriate group exposed to this heavy metal at 1 mg/kg of diet alone or with AME (the Cd5 group vs. the Cd1 group and the Cd5 + AME group vs. the Cd1 + AME group) are marked. Moreover, quantitative differences between the control group and the Cd1, Cd5, Cd1 + AME, and Cd5 + AME groups, as well as between the Cd1 group and Cd5 group and the Cd1 + AME and Cd5 + AME groups, respectively, are presented in figures as a factor of difference (if the values were more than 100% higher or more than 50% lower) or percentage difference (in other cases). The effect size, defined as the strength of difference between the tested groups at each time point and demonstrated as eta squared (η^2^), was calculated. This effect was weak for η^2^ ≤ 0.01, medium for η^2^ between 0.01 and 0.14, and large for η^2^ ≥ 0.14.

Spearman’s correlation test was run to estimate the dependency (positive or negative) between two variables and the degree of the correlation (r). The existence of relationships between the variables assayed in the present study and between these parameters and previously published [17] Cd concentrations in the blood, urine, kidney, and bone was checked. Moreover, to estimate the connection between the impact of Cd and AME on the body homeostasis of Ca and P_i_ and the mineral status and fracture strength of bone, correlations between the variables estimated in this study and previously reported markers of bone metabolism [18] and the main biomechanical indices of bone vulnerability to fracture [19] were evaluated. Spearman’s correlation, based on the value of ǀrǀ, is interpreted as very strong (ǀrǀ > 0.9), strong (0.7 < ǀrǀ ≤ 0.9), moderate (0.4 < ǀrǀ ≤ 0.7), and weak (0.2 ≤ ǀrǀ ≤ 0.4).

## 3. Results

### 3.1. The Impact of Exposure to Cd and/or Supplementation with AME on Ca Concentration in the Serum and Its Urinary Excretion

**Figure 2:**
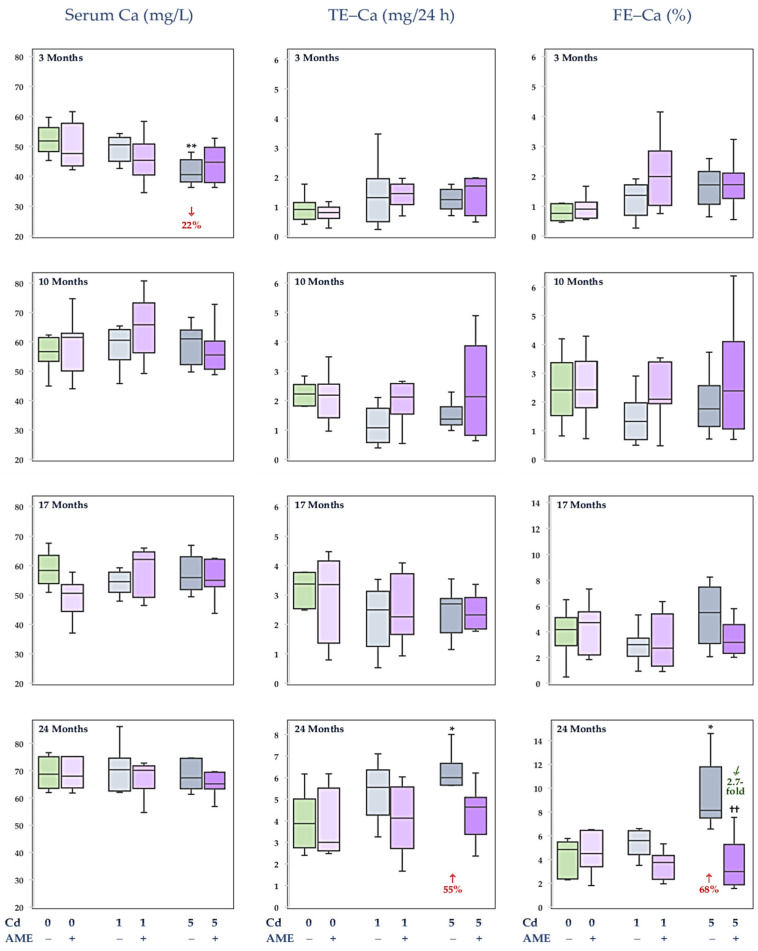
Cadmium (Cd) and/or *Aronia melanocarpa* L. berry extract (AME) effects on calcium (Ca) concentration in the serum and this macroelement’s total (TE–Ca) and fractional (FE–Ca) urinary excretion in female rats. The rodents were exposed to Cd in the Labofeed diet at 0, 1, and 5 mg/kg and/or were supplemented with 0.1% aqueous AME solution administered as the only liquid to drink (“+”, given; “−”, not given). Data represent the median values (the horizontal lines within the bars presenting the confidence interval), 25–75% confidence interval, and the minimum and maximum values for 8 individuals in each group (only after 24 months there were 7 females in the AME, Cd1, and Cd5 groups). Statistically significant differences vs. the control group (* *p* < 0.05 and ** *p* < 0.01) and Cd5 group (^††^ *p* < 0.01) are marked. The numerical values that are evident below or above the bars show a percentage difference or fold of difference in the median values between the two experimental groups (↓, lower vs. the control group; ↑, higher vs. the control group; ↙, lower vs. the Cd5 group). The effect size (η^2^) for the differences in Ca concentration in the serum, TE–Ca, and FE–Ca between the study groups was large (0.222, 0.186, and 0.410, respectively). The numerical values of the serum Ca concentration, TE–Ca, and FE–Ca are available in Table S3.

AME supplementation and/or feeding a diet with added Cd at 1 mg/kg for 3–24 months did not influence the serum Ca concentration and this macroelement’s content in 24 h urine (TE–Ca) and its fractional urinary excretion (FE–Ca) (Figure 2, Table S3). The concentration of Ca in the serum in the female rats on a diet with added Cd at 5 mg/kg was decreased after 3 months (by 22%); however, during the longer treatment, it did not differ in comparison to the control animals (Figure 2; Table S3). TE–Ca and FE–Ca were unaffected in these animals, except for their increased values (by 55% and 68%, respectively) after 24 months. In the group co-treated with Cd at 5 mg/kg of diet and AME (the Cd5 + AME group) for 3–24 months, the serum Ca concentration and urinary excretion of this macroelement (TE–Ca and FE–Ca) reached values within the range of the control group. Moreover, after 24 months of the study, the FE–Ca in the Cd5 + AME group was lower (2.7-fold) compared to the Cd5 group (Figure 2, Table S3).

There were no differences in Ca concentration in the serum and urinary excretion of this macroelement (TE-Ca and FE-Ca) between the Cd1 group and Cd5 group, as well as the Cd1 + AME group and the Cd5 + AME group (Figure 2, Table S3).

### 3.2. The Impact of Exposure to Cd and/or Supplementation with AME on P_i_ Concentration in the Serum and Its Urinary Excretion

**Figure 3:**
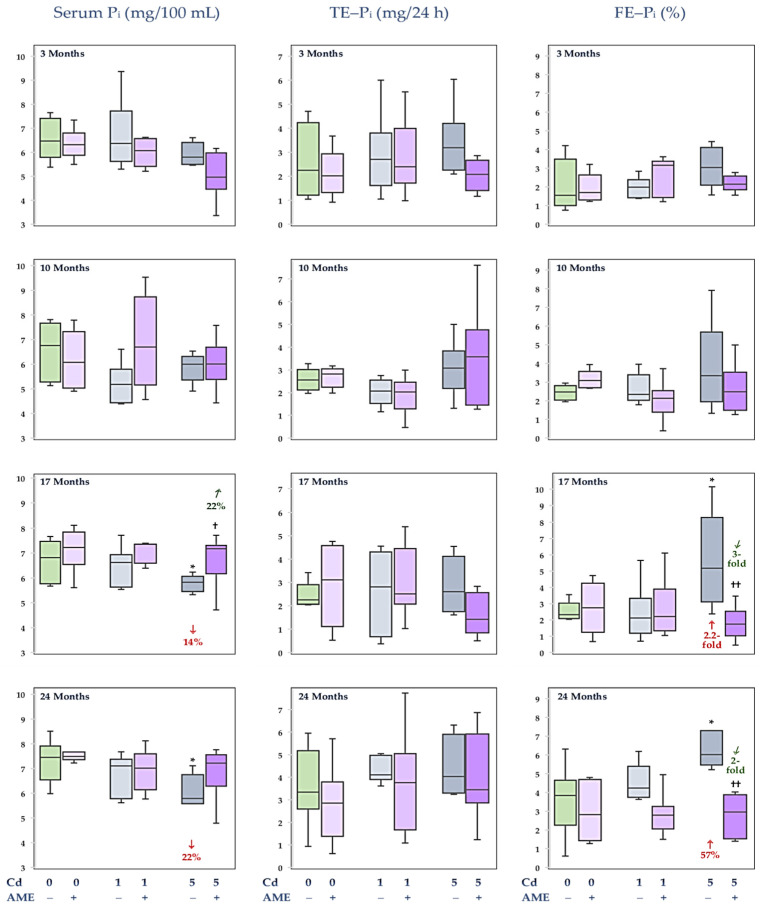
Cadmium (Cd) and/or *Aronia melanocarpa* L. berry extract (AME) effects on inorganic phosphorus (P_i_) concentration in the serum and this microelement’s total (TE–P_i_) and fractional (FE–P_i_) urinary excretion in female rats. The rodents were exposed to Cd in the Labofeed diet at 0, 1, and 5 mg/kg and/or were supplemented with 0.1% aqueous AME solution administered as the only liquid to drink (“+”, given; “−”, not given). Data represent the median values (the horizontal lines within the bars presenting the confidence interval), 25–75% confidence interval, and the minimum and maximum values for 8 individuals in each group (only after 24 months there were 7 females in the AME, Cd1, and Cd5 groups). Statistically significant differences vs. the control group (* *p* < 0.05) and Cd5 group (^†^ *p* < 0.01 and ^††^ *p* < 0.01) are marked. The numerical values that are evident below or above the bars show a percentage difference or fold difference in the median values between the two experimental groups (↓, lower vs. the control group; ↑, higher vs. the control group; ↙, lower vs. the Cd5 group; ↗, higher vs. the Cd5 group). The effect size (η^2^) for the differences in the serum P_i_ concentration (0.216 and 0.235) and FE–P_i_ (0.201 and 0.423) between the study groups was large. The numerical values of the serum P_i_ concentration, TE–P_i_, and FE–P_i_ are available in Table S4.

In the females administered the diet containing Cd at 1 mg/kg and 0.1% AME alone or together throughout the 24-month study, the serum P_i_ concentration and excretion in the urine of this element (TE–P_i_ and FE–P_i_) were within the ranges of values noted in the control group (Figure 3, Table S4). After 17 and 24 months of feeding with the diet with added Cd at 5 mg/kg, P_i_ concentration in the serum was lower (by 14% and 22%, respectively) and the FE–P_i_ was enhanced (2.2-fold and by 57%, respectively) compared to the control animals. The TE–P_i_ was unchanged by the study duration (Figure 3, Table S4). The serum concentration and urinary excretion of P_i_ in the Cd5 + AME group throughout the study did not differ from the control females. In the animals co-administered Cd through the 5 mg/kg diet and AME, the serum P_i_ concentration after 17 months was higher (by 22%), and the FE–P_i_ after 17 and 24 months was lower (3- and 2-fold, respectively) compared to the Cd5 group (Figure 3, Table S4).

There were no differences in the serum concentration of P_i_ and this macroelement loss in urine (TE–P_i_ and FE–P_i_) between the Cd1 and Cd5 groups and the Cd1 + AME and Cd5 + AME groups (Figure 3, Table S4).

### 3.3. The Impact of Exposure to Cd and/or Supplementation with AME on the Serum Concentrations of Calciotropic Hormones

**Figure 4:**
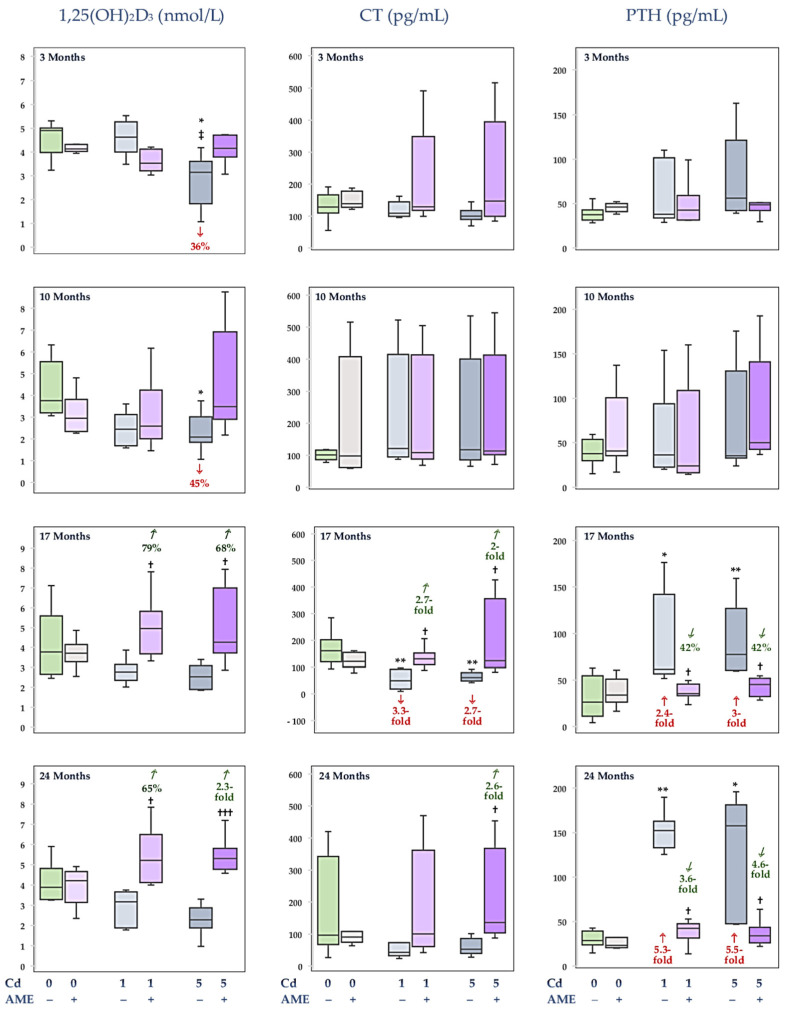
Cadmium (Cd) and/or *Aronia melanocarpa* L. berry extract (AME) effects on the concentrations of 1,25-dihydroxyvitamin D_3_ (1,25(OH)_2_D_3_), calcitonin (CT), and parathormone (PTH) in the serum of female rats. The rodents were exposed to Cd in the Labofeed diet at the concentration of 0, 1, and 5 mg/kg and/or were supplemented with 0.1% aqueous AME solution administered as the only liquid to drink (“+”, given; “−”, not given). Data represent the median values (the horizontal lines within the bars presenting the confidence interval), 25–75% confidence interval, and the minimum and maximum values for 8 individuals in each group (only after 24 months there were 7 females in the AME, Cd1, and Cd5 groups). Statistically significant differences vs. the control group (* *p* < 0.05 and ** *p* < 0.01), the respective group treated with Cd alone (^†^ *p* < 0.05 and ^†††^ *p* < 0.001), and the Cd1 group (^‡^ *p* < 0.05) are marked. The numerical values that are evident below or above the bars show a percentage difference or fold of difference in the median values between the two experimental groups (↓, lower vs. the control group; ↑, higher vs. the control group; ↙, lower vs. the respective Cd group; ↗, higher vs. the respective Cd group). The effect size (η^2^) for the differences in the serum concentrations of 1,25(OH)_2_D_3_, CT, and PTH between the study groups was large (0.235–0.610, 0.383–0.561, and 0.538–0.595, respectively). The numerical values of the concentrations of 1,25(OH)_2_D_3_, CT, and PTH are available in Table S5.

Supplementation with AME alone for up to 24 months did not modify the concentrations of calciotropic hormones in the serum (Figure 4, Table S5). Feeding with the diet containing Cd at 1 mg/kg did not influence 1,25(OH)_2_D_3_ concentration in the serum. After 3 and 10 months of moderate exposure, the concentration of this metabolite of vitamin D_3_ was decreased (by 36% and 45%, respectively) (Figure 4, Table S5). The concentrations of CT and PTH in the serum at both levels of the 3- and 10-month exposure to Cd were within the proper values noted in the control group (Figure 4, Table S5). After 17 months, the concentration of CT in the Cd1 and Cd5 groups was lower (3.3- and 2.7-fold, respectively) than in the control animals. Moreover, PTH concentration at both levels of 17- and 24-month exposure to Cd was higher (from 2.4- to 5.5-fold) than in the control females (Figure 4, Table S5).

AME supplementation under low and moderate exposure to Cd entirely protected against this trace element-induced changes in the concentrations of calciotropic hormones (Figure 4, Table S5). The concentration of 1,25(OH)_2_D_3_ in the serum of the females from the Cd1 + AME and Cd5 + AME groups after 17 and 24 months was higher (by 65% to 2.3-fold) than in the appropriate groups exposed to Cd alone. The 17-month co-administration of Cd and AME resulted in higher (2.7- and 2-fold in the Cd1 + AME group and Cd5 + AME group, respectively) CT concentration in the serum than in the respective groups of animals treated with Cd alone. In the Cd5 + AME group, the concentration of CT in the serum after 24 months was higher (2.6-fold) than in the Cd5 group (Figure 4, Table S5). The concentration of PTH in the serum after 17 and 24 months of the application of AME during the animals’ maintenance on diets containing Cd at 1 and 5 mg/kg was lower (by 42% to 4.6-fold) than in the appropriate groups that received this trace metal alone (Figure 4, Table S5).

There were no differences in the concentrations of calciotropic hormones in the serum between the Cd1 and Cd5 groups and the Cd1 + AME and Cd5 + AME groups throughout the study, except for lower (by 32%) 1,25(OH)_2_D_3_ concentration in the Cd5 group than in the Cd1 group after 3 months (Figure 4, Table S5).

### 3.4. The Impact of Exposure to Cd and/or Supplementation with AME on Klotho Concentration in the Serum

**Figure 5:**
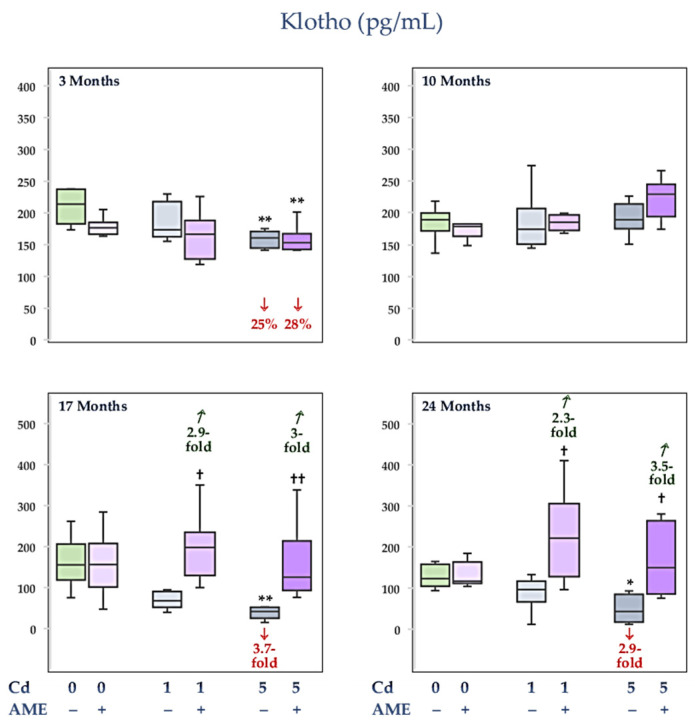
Cadmium (Cd) and/or *Aronia melanocarpa* L. berry extract (AME) effects on the serum concentration of Klotho in female rats. The animals were exposed to Cd in the Labofeed diet at the concentration of 0, 1, and 5 mg/kg and/or were supplemented with 0.1% aqueous AME solution administered as the only liquid to drink (“+”, given; “−”, not given). Data represent the median values (the horizontal lines within the bars presenting the confidence interval), 25–75% confidence interval, and the minimum and maximum values for 8 individuals in each group (only after 24 months there were 7 females in the AME, Cd1, and Cd5 groups). Statistically significant differences vs. the control group (* *p* < 0.05 and ** *p* < 0.01) and the respective group treated with Cd alone (^†^ *p* < 0.05 and ^††^ *p* < 0.01) are marked. The numerical values that are evident below or above the bars show a percentage difference or fold of difference in the median values between the two experimental groups (↓, lower vs. the control group; ↗, higher vs. the respective Cd group). The effect size (η^2^) for the differences in the serum concentrations of Klotho between the study groups was large (0.331–0.577). The numerical values of the concentration of Klotho are available in Table S6.

Being supplied with AME and treatment with Cd at 1 mg/kg of diet alone and together for the whole experiment had no impact on Klotho concentration in the serum; however, in the Cd1 + AME group, the concentration after 17 and 24 months was higher (2.9- and 2.3-fold, respectively) than in the Cd1 group (Figure 5, Table S6). Feeding the diet containing Cd at 5 mg/kg caused a drop (25% to 3.7-fold) in this protein concentration, except for its unchanged level after the 10-month exposure duration (Figure 5, Table S6). In the animals that received AME during the moderate Cd treatment for 3 months, the concentration of Klotho was decreased (by 28%) to a similar extent as in the Cd5 group. However, 17- and 24-month AME supplementation during feeding with the diet containing Cd at 5 mg/kg prevented a reduction in the concentration of this protein caused by this trace element. The concentration of Klotho in the Cd5 + AME group was within the range of the control group and was higher (3- and 3.5-fold after 17 and 24 months, respectively) than in the Cd5 group (Figure 5, Table S6).

The serum concentration of Klotho did not differ between the Cd1 and Cd5 groups and the Cd1 + AME and Cd5 + AME groups (Figure 5, Table S6).

### 3.5. The Impact of Exposure to Cd and/or Supplementation with AME on the Concentrations of 1alpha-OHase and 1,25(OH)_2_D_3_ in the Kidney

**Figure 6:**
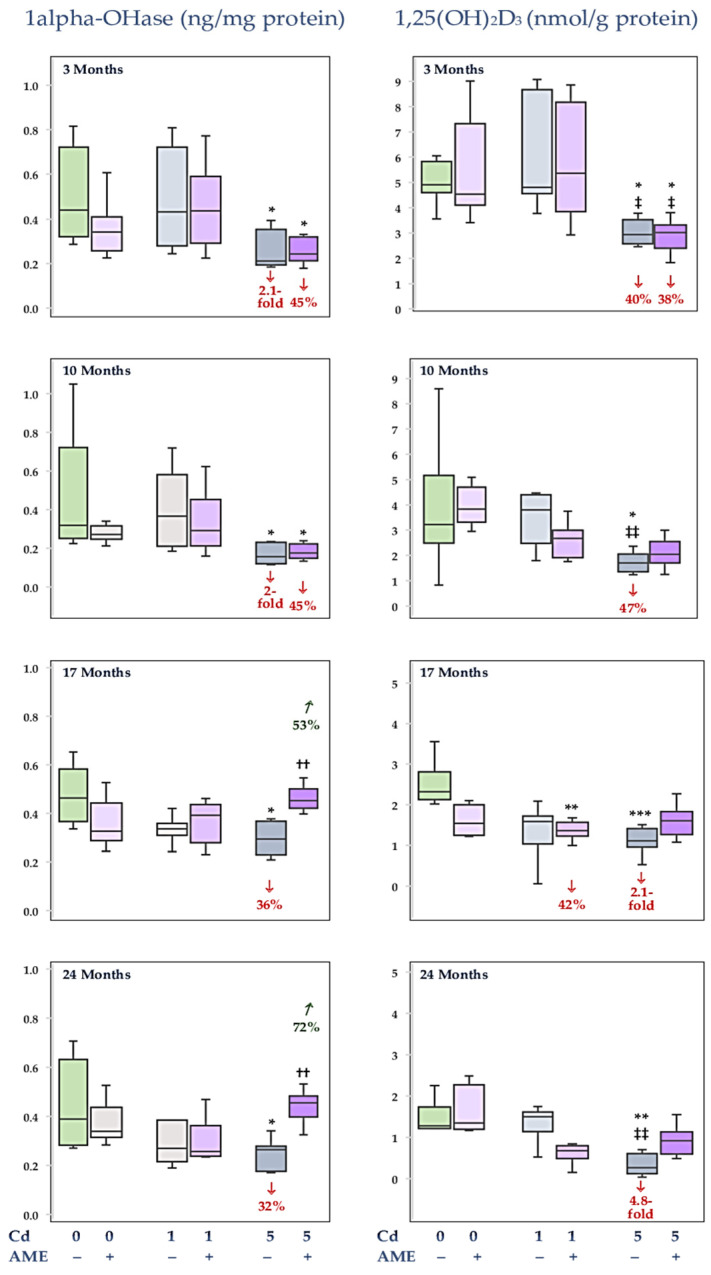
Cadmium (Cd) and/or *Aronia melanocarpa* L. berry extract (AME) effects on the concentrations of 25-hydroxyvitamin D 1alpha-hydroxylase (1alpha-OHase) and 1,25-dihydroxyvitamin D_3_ (1,25(OH)_2_D_3_) in the kidney of female rats. The rodents were exposed to Cd in the Labofeed diet at the concentration of 0, 1, and 5 mg/kg and/or were supplemented with 0.1% aqueous AME solution administered as the only liquid to drink (“+”, given; “−”, not given). Data represent the median values (the horizontal lines within the bars presenting the confidence interval), 25–75% confidence interval, and the minimum and maximum values for 8 individuals in each group (only after 24 months there were 7 females in the AME, Cd1, and Cd5 groups). Statistically significant differences vs. the control group (* *p* < 0.05, ** *p* < 0.01, and *** *p* < 0.001), Cd5 group (^††^ *p* < 0.01), and the respective group receiving Cd at 1 mg/kg of diet alone (Cd1 group) or with the AME (Cd1 + AME group) (^‡^ *p* < 0.05, ^‡‡^ *p* < 0.01) are marked. The numerical values that are evident below or above the bars show a percentage difference or fold of a difference in the median values between the two experimental groups (↓, lower vs. the control group; ↗, higher vs. the Cd5 group). The effect size (η^2^) for the differences in the kidney concentrations of 1alpha-OHase and 1,25(OH)_2_D_3_ between the study groups was large (0.298–0.360 and 0.433–0.532, respectively). The numerical values of the concentrations of 1alpha-OHase and 1,25(OH)_2_D_3_ are available in Table S7.

The administration of AME and the diet with Cd added at 1 mg/kg alone and in conjunction for 3–24 months did not influence the kidney concentrations of 1alpha-OHase and 1,25(OH)_2_D_3_, except for a lower (by 42%) concentration of 1,25(OH)_2_D_3_ due to the 17-month study in the Cd1 + AME group (Figure 6, Table S7). At the exposure to Cd at 5 mg/kg of diet, the concentrations of 1alpha-OHase and 1,25(OH)_2_D_3_ at each time point were lower (by 32% to 2.1-fold and 40% to 4.8-fold, respectively) (Figure 6, Table S7). The kidney concentration of 1alpha-OHase in the animals supplemented with AME under the 3- and 10-month treatment with Cd at 5 mg/kg of diet was lower (by 45%) than in the control group and did not differ from the Cd5 group. However, after the longer study duration (17 and 24 months), the concentration was within the range of values determined in the control group and reached higher values (by 53% and 72%, respectively) than in the Cd5 group (Figure 6, Table S7). The kidney concentration of 1,25(OH)_2_D_3_ in the females that received AME during 3-month intoxication with Cd at 5 mg/kg of diet was lower (by 38%) than in the control group and was within the range of the Cd5 group. After 10, 17, and 24 months, the kidney concentration of this active metabolite of vitamin D_3_ in the Cd5 + AME group reached values determined in the control group and it did not differ from the concentration assayed in the Cd5 group (Figure 6, Table S7).

There were no differences in the concentration of 1alpha-OHase between the Cd1 and Cd5 groups and the Cd1 + AME and Cd5 + AME groups throughout the study. The kidney concentration of 1,25(OH)_2_D_3_ after 3-, 10-, and 24-month moderate exposure to Cd was lower (by 39%, 2.2-fold, and 5.6-fold) than after the low-level treatment. Moreover, after the 3-month study, the concentration of this active metabolite of vitamin D_3_ in the Cd5 + AME group was lower (by 44%) than in the Cd1 + AME group (Figure 6, Table S7).

### 3.6. Relationships Between Indices of Ca and P_i_ Metabolism and Markers of the Pathways of Its Regulation

Numerous positive and negative relationships between some of the examined indices of the metabolism of Ca and P_i_ and markers of the pathways of its regulation in the female rats not supplemented or supplemented with AME were noted (Table 2). The serum concentration of Ca in the females that received no supplementation with AME (the control group, Cd1 group, and Cd5 group) negatively correlated with the serum concentrations of CT and Klotho and the serum and kidney concentration of 1,25(OH)_2_D_3_ and positively with TE–Ca and FE–Ca. Moreover, the concentration of 1,25(OH)_2_D_3_ in the serum of these animals positively correlated with the concentrations of 1alpha-OHase and 1,25(OH)_2_D_3_ in the kidney. The concentration of Klotho in the serum was positively related to the concentrations of 1,25(OH)_2_D_3_ and CT and negatively with PTH concentration. Positive dependencies were also noted between the concentrations of 1alpha-OHase and 1,25(OH)_2_D_3_ in the kidney and the concentrations of CT and Klotho in the serum. An inverse dependency was noted between PTH concentration in the serum and the concentrations of 1alpha-OHase and 1,25(OH)_2_D_3_ in the kidney. In the rodents that did not receive AME, a positive relationship was noted between P_i_ and 1,25(OH)_2_D_3_ concentrations in the serum (Table 2).

Positive relationships were noted between the serum concentration of Ca and the serum concentration of 1,25(OH)_2_D_3_ and TE–Ca in the females administered AME. There was no relationship between the concentrations of 1,25(OH)_2_D_3_ in the serum and 1alpha-OHase in the kidneys of these animals. A negative correlation was revealed between the serum and kidney concentration of this active vitamin D_3_ metabolite. Moreover, an inverse relationship occurred between the serum concentration of Ca and the kidney concentration of 1,25(OH)_2_D_3_ and the serum concentration of CT positively correlated with the concentration of 1alpha-OHase in the kidney (Table 2).

A positive relationship occurred between the serum concentrations of Ca and P_i_ in the animals administered AME or not receiving the supplementation. Moreover, no association between the serum concentration of P_i_ and its urinary excretion (TE–P_i_ and FE–P_i_) was noted in these animals (Table 2).

### 3.7. Relationships Between the Determined Indices of the Metabolism of Ca and P_i_ and Markers of the Pathways of Its Regulation and Cd Content in the Body

In the rodents that did not receive AME supplementation (the control animals, Cd1 group, and Cd5 group), negative associations between the concentrations of P_i_, CT, 1,25(OH)_2_D_3_, and Klotho in the serum, as well as the kidney concentrations of 1alpha-OHase and 1,25(OH)_2_D_3_ and the main markers of Cd content in the body (i.e., this element concentration in the blood, kidney, and urine) were noted (except for no dependence between the serum P_i_ concentration and the kidney Cd concentration) (Table 3). PTH concentration in the serum and renal P_i_ handling (TE–P_i_ and FE–P_i_) were positively associated with Cd in the blood, kidney, and urine, excepting a lack of correlation of TE–P_i_ with blood Cd (Table 3). There were no dependences between the indices of Cd body burden and Ca concentration in the serum and its urinary excretion, except for positive relationships between FE–Ca and Cd blood and kidney concentrations (Table 3).

In the AME-supplemented females (the AME group, Cd1 + AME group, and Cd5 + AME group), the serum and kidney concentrations of 1,25(OH)_2_D_3_ correlated positively and negatively, respectively, with Cd concentrations in the blood, kidney, and urine (Table 3). There were no other associations between the determined variables and the markers of Cd content in the body of these animals (Table 3).

### 3.8. Relationships Between the Determined Indices of the Metabolism of Ca and P_i_ and Markers of the Pathways of Its Regulation and Indices of the Bone and Kidney Status

In the rodents used in our study, and especially in those not supplemented with AME (the control animals, Cd1 group, and Cd5 group), numerous dependencies occurred between the determined indices of the metabolism of Ca and P_i_ and markers of the investigated pathways of its regulation, as well as markers of the bone and kidney status (Table 4, Table 5, Table 6 and Table 7).

It is important to emphasise that in the animals not supplemented with AME, negative dependencies occurred between the indices of bone mineral status, i.e., the percentage content of minerals and Ca concentration in the distal femoral epiphysis bone tissue, serum Ca and PTH concentrations, and renal Ca and P_i_ handling (Table 4). Ca and P_i_ concentrations in the serum of these females positively correlated with the femoral diaphysis strength (yield and fracture strength) (Table 4). Moreover, the concentrations of CT, 1,25(OH)_2_D_3_, and Klotho in the serum and the kidney concentrations of 1,25(OH)_2_D_3_ and 1alpha-OHase were negatively related to numerous biomarkers of Cd nephrotoxicity determined in the urine (kidney injury molecule-1—KIM-1, *N*-acetyl-β-D-glucosaminidase—NAG, β2-microglobulin—β2-MG, alkaline phosphatase—ALP, albumin concentration in the urine adjusted for creatinine—ACR, and total protein concentration in the urine adjusted for creatinine—PCR) (Table 6). Ca and PTH concentrations in the serum and urinary excretion of Ca and P_i_ positively correlated with numerous biomarkers of kidney function (Table 6).

In the animals that received AME, Ca concentration in the serum was inversely related to the percentage of minerals and Ca concentration in the distal femoral epiphysis bone tissue, and a negative dependency occurred between the serum P_i_ and the percentage content of minerals in the distal epiphysis of the femur (Table 5). In the female rats supplemented with AME, similarly to those not given this extract, some positive correlations were noted between the markers of kidney damage and Ca and P_i_ concentrations in the serum and their renal handling (Table 7). Moreover, negative dependencies were revealed between 1,25(OH)_2_D_3_ concentration in the kidney and KIM-1, β2-MG, NAG, ALP, ACR, and PCR in the urine (Table 7).

## 4. Discussion

This article is the first focused on the investigation, in an animal model of current environmental human exposure to Cd, of the unfavourable impact of this toxic element and the beneficial influence of supplementation with AME on the body homeostasis of Ca and P_i,_ including the main pathways of its regulation. This study not only revealed the destroying dose-dependent effect of Cd and the protective impact of AME on Ca and P_i_ metabolism but also provided important findings for the explanation of the possible mechanisms of the previously reported damage to the skeletons of these animals under low-level and moderate intoxication with Cd and the beneficial influence of the supplementation with AME [18,19,20].

The body status of Ca and P_i_ undergoes strict regulation through a complex axis including the intestine, bone, kidney, thyroid C-cells, and parathyroids [34,41,44]. Calciotropic hormones (PTH, CT, and 1,25(OH)_2_D_3_), Klotho, and fibroblast growth factor 23 (FGF-23), as well as these macroelements’ concentrations in the serum and their intake in the diet, are involved in the regulation of these minerals’ homeostasis [37,41,44,48]. In the case of a decrease in the serum Ca concentration, PTH release by parathyroids increases and, in turn, the resorption of this element from the skeleton is enhanced due to the stimulation of osteoclasts to resorb bone. Moreover, PTH increases this element’s reabsorption in the kidney tubules and enhances (via activation of 1alpha-OHase) the production of 1,25(OH)_2_D_3_ in the kidney mitochondria. This active vitamin D_3_ metabolite is necessary for the biosynthesis of Ca-binding protein (Ca–BP) in the intestine, which is responsible for gastrointestinal Ca absorption. As a result, the serum Ca concentration normalises due to its enhanced absorption from the digestive tract and release from bone and decreased urinary loss [37,41]. An enhancement in Ca and 1,25(OH)_2_D_3_ concentrations in the serum limits PTH secretion via parathyroid glands. CT has the opposite action to that of PTH. It lowers Ca concentration in the serum via inhibiting osteoclastic bone resorption [37,41]. The serum concentration of P_i_ is mainly regulated by 1,25(OH)_2_D_3_, CT, PTH, Klotho, FGF-23, and phosphate intake [34,41,43,44]. Analogously to the case of Ca, 1,25(OH)_2_D_3_ enhances the serum phosphate concentration due to increasing their intestinal absorption, tubular reabsorption, and release from bone [37,41]. PTH and Klotho inhibit phosphates’ tubular reabsorption, decreasing their serum concentration [41,43,44]. Thus, to estimate Ca and P_i_ homeostasis in the body, the serum concentration and renal handling of these elements and the main regulators of their metabolism in the serum (PTH, 1,25(OH)_2_D_3_, CT, and Klotho), as well as bone mineral status, should be evaluated, as was performed in our study, the results of which are the subject of the present paper or have already been published [18].

The unchanged serum concentrations of Ca and P_i_ and their proper total and fractional urinary excretion, as well as the unaffected vitamin D_3_ metabolism in the female rats, maintained during the study on a diet containing an addition of Cd at 1 mg/kg, could suggest that even long-term low-level exposure had no destroying impact on the body status of these minerals. However, such a conclusion is unfounded because we have recently reported that exposure negatively influenced the skeleton mineral status already after a 10-month duration (decreased femoral BMD and the percentage content of minerals and Ca concentration in the distal femoral epiphysis) and that this effect persisted throughout the entire experiment [18]. The measurements of biomarkers of bone turnover in these animals revealed that treatment with Cd inhibited bone formation (reduced the serum and bone tissue ALP activity and lower concentrations of osteocalcin—OC—in the serum, osteoprotegerin—OPG—in the serum and bone, and procollagen I—PC I—in the bone) and enhanced bone resorption (increased concentrations of carboxy-terminal cross-linking telopeptides of type I collagen—CTX—and soluble receptor activator of nuclear factor-κB ligand—sRANKL—in the bone tissue and/or serum) (Table S1) [18,19]. The previously revealed increased bone resorption in the female rats already after 3 months and poorer bone mineralization starting from the 10-month low-level Cd treatment [18], shown in the current investigation, increased the serum concentration of PTH after 17 and 24 months and allowed us to conclude that the proper Ca and P_i_ concentrations in the serum of these animals might result from enhanced release of these elements from the skeleton. The demonstration that feeding a diet containing Cd at 1 mg/kg negatively affected the bone mineral status compared to proper calcaemia and phosphataemia and did not affect the renal handling of these elements is a significant finding of practical importance because it shows that the assay of the concentrations of Ca and P_i_ in the serum and urine at low exposure to Cd may be insufficient to detect an early unfavourable effect of this xenobiotic on the mineral metabolism, and the measurement of BMD together with markers of bone turnover should be performed for this purpose. The negative correlations between the serum Ca concentration and the percentage content of minerals and this element’s concentration in the distal epiphysis of the femur, the inverse relationships among the serum concentration of PTH and markers of bone mineralisation (BMD of the femur and mineral components’ percentage content and the Ca concentration in the bone at the distal epiphysis of this long bone), and the positive relationship between the serum Ca and P_i_ seem to confirm our theory of the release of Ca and P_i_ from the skeleton to maintain calcaemia and phosphataemia at the proper level during low Cd exposure.

It is worth emphasising that although the values of the determined parameters did not differ in the Cd1 and Cd5 groups (except for the lower concentration of 1,25(OH)_2_D_3_ in the serum and kidney at some time points during the higher treatment), it should be concluded that the effect of Cd on Ca and P_i_ homeostasis and its regulation by calciotropic hormones and Klotho differed between the two levels of exposure. The decreased serum concentrations of Ca after 3-month treatment and P_i_ after 17- and 24-month treatment with Cd at 5 mg/kg of diet (Cd5 group) show that the mechanisms responsible for regulating calcaemia and phosphataemia were affected due to the moderate exposure to this xenobiotic. The decreased Ca concentration in the serum was the only evidence of its disrupted homeostasis caused by feeding for 3 months on a diet containing Cd at 5 mg/kg. The renal handling of Ca at this time point was proper and the previously reported [18] concentration of this element in the distal epiphysis of the femur and femoral BMD were within the values determined in the control animals. Because the concentration of mitochondrial 1alpha-OHase in the kidney and 1,25(OH)_2_D_3_ in the serum and kidney were decreased after 3 months of treatment with Cd at 5 mg/kg of diet, hypocalcaemia caused by this toxic element (at enhanced sRANKL concentration in the bone tissue and serum indicating enhanced bone resorption [17]) may be explained by insufficient gastrointestinal absorption of Ca. As was revealed in our study and confirmed by negative dependencies between Cd concentration in the kidney and the measured indicators of vitamin D_3_ metabolism, accumulation of this toxic heavy metal in the kidneys resulted in the inhibition of 1alpha-OHase, responsible for 25-hydroxyvitamin D_3_ (25(OH)D_3_) hydroxylation to 1,25(OH)_2_D_3_ in the mitochondria. This only biologically active vitamin D_3_ metabolite is needed for the intestinal synthesis of Ca–BP, which is responsible for gastrointestinal Ca absorption [35]. Moreover, Cd divalent ions (Cd^2+^), because of their similarity to Ca ions (Ca^2+^), may compete with ions of this macroelement for binding into Ca–BP and, in this way, also decrease Ca absorption [31].

Because the skeleton is the main place of Ca and P_i_ storage in the body, there is a close relationship between these elements’ homeostasis and the bone mineral status and biomechanical strength [29,30,32]. A drop in the concentrations of these minerals in the extracellular fluid results in their enhanced release from the skeleton to normalise calcaemia and phosphataemia. That is why, the disruption of Ca and P_i_ homeostasis leads to changes in the bone mineral status, as was noted in our study, due to low-to-moderate treatment with Cd (Table S1) [18] and higher intoxication [29,30]. In previous research [31], we found that the administration of Cd in drinking water at the concentration of 1 mg/L (daily Cd intake at its administration in drinking water is higher than at the treatment at the same concentration in a diet) for 24 months decreased the concentrations of vitamin D_3_ metabolites (25(OH)D_3_ and 1,25(OH)_2_D_3_) and CT in the serum and the tabular reabsorption of Ca and P_i_ and increased the serum PTH concentration without influencing calcaemia and phosphataemia. These changes led to a decreased content of mineral components in the bone tissue, including Ca and phosphates, resulting in vertebral osteopenia or osteoporosis in female rats [29]. The administration of Cd at concentration of 5 and 50 mg/L of drinking water (but not at 1 mg/L) for one year was the cause of lower content of Ca, phosphate, and total mineral components in the lumbar vertebral body of male rats [30]. The treatment influenced the serum concentrations of calciotropic hormones (1,25(OH)_2_D_3_, 25(OH)D_3_, and CT concentrations were decreased and that of PTH was increased). Moreover, it enhanced FE–Ca and FE–P_i_ without impacting the serum concentrations of both elements [30]. Decreased serum concentrations of 1,25(OH)_2_D_3_ and CT and increased FE–P_i_ were also noted due to intoxication with Cd at 1 mg/L of drinking water [30]. Hypocalcaemia and proper serum P_i_ concentration were revealed in laying hens maintained on a feed with Cd at 60.67 mg/kg [49]. Abnormal concentrations of calciotropic hormones (decreased 1,25(OH)_2_D_3_ and CT concentrations and increased PTH concentration) were noted at almost 4- or 2-fold lower Cd concentrations in the diet (15.56 mg/kg or/and 30.55 mg/kg) than that at which hypocalcaemia occurred (60.67 mg/kg) but not at 0.47 and 7.58 mg/kg [49]. Yuan et al. [50] noted that mixed sub-chronic intoxication with Cd and lead (Pb) led to a decrease in the serum concentrations of Ca and P_i_, an increase in these elements’ concentration in the urine, and to destroying bone metabolism.

The negative dependencies between the serum concentration of Ca and the percentage content of minerals in the bone, together with increased bone resorption, allowed for the conclusion that the proper concentration of this element in the serum at both levels of Cd exposure throughout the whole study, except for its decreased level after the 3-month moderate treatment, might result from the enhanced bone resorption noted in these female rats [18]. The adverse relationships between the markers of Ca and P_i_ renal handling, the mineral content in the distal femoral epiphysis bone tissue, and the bone Ca concentration confirm that the excessive urinary loss of these elements was a cause of the lower bone mineralisation noted in these animals.

The unaffected concentrations of 1alpha-OHase and 1,25(OH)_2_D_3_ maintained throughout the study by females on a diet with added Cd at 1 mg/kg showed that low exposure that is capable of destroying the bone tissue metabolism might not influence vitamin D_3_ metabolism. However, at the moderate treatment (5 mg/kg of diet), due to the higher content of this xenobiotic in the body (including its higher accumulation in the kidney) than at the exposure to Cd at 1 mg/kg of diet, the concentration of 1,25(OH)_2_D_3_ in the serum was decreased, already after 3 months, due to its reduced synthesis in the way of hydroxylation of 25(OH)D in the kidney mitochondria, as it was revealed based on the lower kidney concentrations of 1alpha-OHase and 1,25(OH)_2_D_3_. The negative relationships between Cd concentration in the kidney and the concentrations of 1,25(OH)_2_D_3_ and 1alpha-OHase in the serum and/or kidney confirm this mechanism. The unchanged 1,25(OH)_2_D_3_ concentration in the serum after 17- and 24-month treatment with Cd at 5 mg/kg of diet, together with the lower extent of inhibition of 1alpha-OHase than after 3 and 10 months, may be explained by the stimulation of the renal synthesis of 1,25(OH)_2_D_3_ to maintain calcaemia. After 3 and 10 months, when the serum concentration of 1,25(OH)_2_D_3_ was decreased, the kidney concentration of 1alpha-OHase was lower by about 2-fold, whereas, after 17 and 24 months, at the proper serum concentration of this active vitamin D_3_ metabolite, the kidney concentration of this enzyme was decreased to a lower extent (by 36% and 32%, respectively). Moreover, the kidney 1,25(OH)_2_D_3_ concentration was decreased more markedly than earlier. Thus, it seems possible that due to the lower Ca concentration in the serum in the first stage of moderate exposure, homeostatic mechanisms, including mainly the release of this element from bone tissue, were activated to compensate calcaemia. That is why the concentration of this macroelement in the serum was proper after longer exposure despite its enhanced urinary loss.

Since the kidney plays a crucial role in the regulation of vitamin D_3_ metabolism and the maintenance of Ca and P_i_ homeostasis [34,35,43,49], the destruction of the body status of these minerals due to exposure to Cd as revealed in the present study might also result from the injurious impact of this toxic element on this organ already reported by us in these females [21]. Both low-level and moderate intoxication with this heavy metal led to abnormalities in the morphological structure and function of the renal tubules and glomeruli [21]. The first sign of Cd nephrotoxicity at both levels of exposure was an elevation in the concentration of KIM-1 in the urine, already after 3 months, indicating damage to the tubules. The damage gradually progressed, especially at moderate exposure (changes in commonly considered sensitive markers of this heavy metal’s nephrotoxicity—β2-MG concentration and NAG activity—occurred a few to several months later than the increase in KIM-1 concentration). Thus, the increased urinary excretion of Ca (TE–Ca and FE–Ca after 24 months) and P_i_ (FE–P_i_ after 17 and 24 months) due to the moderate exposure to Cd may be explained by the injurious effect of this trace element on the kidney, including the decreased tubular reabsorption noted in these animals [21]. The positive dependencies between the markers of these bioelements’ renal handling (total daily and fractional excretion) and indices of tubular damage (NAG, β2-MG, KIM-1, and ACR) in rats not receiving AME supplementation (the control group, Cd1 group, and Cd5 group) confirmed the existence of a relationship between Cd-caused tubular damage and decreased bone mineral status.

The important result of the present study is the finding that the biosynthesis of the only active metabolite of vitamin D_3_ may be disrupted by Cd at its low concentration in the kidney mitochondria, reaching 0.974–1.672 μg/g (mean 1.362 μg/g; such concentrations were determined in the Cd5 group after 3-month exposure) [22]. The role of 1,25(OH)_2_D_3_ in the body is not limited to the regulation of mineral metabolism and influencing bone health, but it also includes, among others, immunological functions [51]. Thus, some of the harmful outcomes of Cd after moderate treatment may be related to 1,25(OH)_2_D_3_ deficiency due to its destroyed renal biosynthesis. The numerous negative correlations between the kidney concentrations of 1,25(OH)_2_D_3_ and 1alpha-OHase and the markers of kidney injury (β2-MG, NAG, KIM-1, ALP, ACR, and PCR) noted in rats not administered AME, together with negative dependencies between these two indices of vitamin D_3_ metabolism and Cd concentration in the blood, urine, and kidney, show that Cd accumulation in the kidney and its damaging impact on this organ resulted in destroying vitamin D_3_ metabolism. In our study, the metabolism of this vitamin was destroyed at Cd concentrations in the blood and urine reaching 0.735–1.332 μg/L and 0.285–0.695 μg/g creatinine, respectively [17], whereas, at the lower concentrations noted in the Cd1 group (0.103–0.324 μg/L and 0.085–0.285 μg/g creatinine, respectively [17]), it was unaffected. Our findings are in line with those of Engström et al. [52], who reported that 1,25(OH)_2_D_3_ concentration in the serum was proper in women with a low concentration of Cd in the blood (0.13–0.72 μg/L; median 0.24 μg/L) and urine (0.14–0.39 μg/L; median 0.25 μg/L) despite reduced BMD and increased bone resorption. Chwalba et al. [36] noted that children with a Cd concentration in the blood above 0.27 μg/L had a lower serum concentration of 25(OH)D_3_ than those having this toxic element at a concentration below 0.27 μg/L. Chen et al. [53] revealed that subjects with high concentrations of this metabolite of vitamin D_3_ in the serum were at a lower risk of kidney dysfunction induced by Cd than those with its insufficiency.

The findings of the current research, analysed together with our previously reported outcomes in the same females [18,19,20], provided important data on the probable pathways of the toxic impact of Cd on the bone at low and moderate treatments. The primary mechanism of Cd osteotoxicity seems to be its direct impact on the metabolism of bone tissue via mediating the activity of osteoblasts and osteoclasts [18]. However, the Cd-induced destruction of Ca and P_i_ balance in the body, changed calciotropic hormone concentrations in the serum, and the decreased concentrations of 1alpha-OHase and 1,25(OH)_2_D_3_ in the kidney allowed us to reach the conclusion that this heavy metal, especially at moderate exposure, might also stimulate the resorption of bone tissue via an indirect mechanism consisting of destroying the body mineral status. The numerous correlations noted between the indices of Ca and P_i_ body status_,_ as well as calciotropic hormone concentrations and the recently reported markers of bone metabolism and biomechanical properties, confirmed a relationship between the disorders in the metabolism of the main bone essential minerals and the weakening of the bone strength properties caused by Cd.

Because our experimental model reflects current low-to-moderate environmental exposure to Cd well [4,8,11,12,13,17], the findings of [17,18,19,20,21,22,23], including the results of the present study, can be extrapolated to women. Our previous studies revealed the unfavourable effect of even relatively low chronic treatment with Cd on the skeleton and organs involved in regulating bone metabolism, such as the kidneys and liver [17,18,19,20,21,22]. Since, in the general population, similar Cd concentrations in the blood and urine were reported [4,8,11,12,13] as the concentrations at which the disruption of Ca and P_i_ homeostasis and its regulation via calciotropic hormones and Klotho were noted in the present study, the extrapolation of these results to the general population shows that women may be at risk of disorders in mineral metabolism due to chronic, mainly moderate, environmental exposure to Cd. However, further epidemiological studies are still necessary to better understand the risk of skeleton injury at an environmental exposure to this toxic element.

The key new achievement of the current study, with practical implications, is the finding that the co-administration of AME during long-term dietary exposure to Cd improved the pathways involved in the regulation of the balance of Ca and P_i_ in the body and allowed maintaining the concentrations of these elements in the serum and their urinary excretion, as well as the concentrations of calciotropic hormones and Klotho at the proper levels. This study not only revealed the beneficial effect of supplementation with AME on Ca and P_i_ homeostasis but also contributed to better explaining the possible mechanisms of the previously reported [18,19,20] favourable impact of the extract on the skeleton under treatment with this heavy metal.

The beneficial effect of supplementation with AME on Ca and P_i_ homeostasis and the pathways of its regulation can be explained by the indirect and direct action of the extract ingredients. The indirect mechanism seems to be related to the formation by polyphenols of stable complexes with Cd^2+^, resulting in the reduction of this heavy metal’s gastrointestinal absorption and, in turn, lower Cd storage in the body, as was reported by us in these female rats [17,22]. Because the extract administration protected from Cd retention in the body, including the skeleton and kidneys [17,22], at the same time, it attenuated the effect of its direct impact, including destroying the bone metabolism and strength properties [18,19,20] and kidney function [21] as it was already reported in these animals [18,19,20,21], protecting from Cd-caused destruction of Ca and P_i_ metabolism and, in turn, from its negative impact on the skeleton. The total protection demonstrated in the currently reported investigation against decreases in the concentrations of 1alpha-OHase and 1,25(OH)_2_D_3_ in the kidney and, subsequently, also the serum concentration of this active vitamin D_3_ metabolite at moderate exposure to this toxic element might result, at least partially, from the previously reported lower retention of this trace element in the kidney, including the mitochondria, in which this active metabolite of vitamin D_3_ is generated, due to the concomitant administration of AME [17,22].

The protective effect of AME might also result from the direct action of its ingredients, apart from the above-described indirect mechanism. Considering the composition of AME, it is reasonable to state that the direct favourable effect of the extract also resulted from the direct action of its ingredients, mainly including polyphenols. At the research stage we are currently at, it is difficult to clarify which components of aronia berries were responsible for the direct effect; however, it seems likely that this action could be mediated by polyphenolic compounds, in which chokeberries are rich [18,47]. Polyphenols and products abundant in these compounds are well known for their favourable action in the skeleton, and their consumption is widely recommended as a strategy for improving bone health [54,55,56,57,58,59,60]. There is no data in the literature on the influence of products based on *A. melanocarpa* berries on the metabolism of minerals and bone; however, it has been shown that some ingredients of chokeberry extract, such as quercetin [61,62,63,64], chlorogenic acid [65], and cyanidin-3-glucoside [66], may have a favourable impact on mineral and bone metabolism. Quercetin has been reported to effectively prevent the decrease in Ca absorption in the intestines caused by substances that deplete reduced glutathione (GSH), including menadione. However, this compound, when administered alone, did not modify this bioelement’s absorption in chickens [62]. Cd is a prooxidant agent, and it was noted to induce oxidative stress due to the low-to-moderate exposure in some organs and tissues with GSH depletion, including bone tissue [20,23]. Thus, it seems that quercetin, via its antioxidative and anti-inflammatory properties [62], may directly protect bone from the prooxidative action of Cd in bone tissue. Other aronia berry ingredients, including bioelements and vitamins, were also reported to influence the skeleton positively [57,59]. Unfortunately, the influence of these substances on mineral metabolism and bone status under treatment with Cd has not been examined so far. It has been revealed that moderate consumption (<1 cup per day) of blueberries, which are a rich source of polyphenols (anthocyanins, flavonols, and chlorogenic acid), may be effective in the attenuation of accelerated bone loss in healthy postmenopausal women [58]. Cladis et al. [67] reported that the administration of purified blueberry polyphenols to ovariectomised rats in a high dose (1000 mg/kg b.wt.) increased Ca absorption. In our study, we have not evaluated the possible protective impact of other polyphenol-rich food products or supplements regarding the harmful effects of exposure to Cd. We were focused on the comprehensive investigation of the possibility of using AME to counteract the effects of low-to-moderate chronic exposure to this trace element. However, the revealed effectiveness of the extract against the numerous outcomes of Cd toxicity [17,18,19,20,21,22,23] may suggest that other polyphenol-rich food products should also be examined as potential protective agents.

The results of the present study, together with our previously reported findings in this experimental model [18,19,20,21,22,23], show that the daily consumption of AME at a dose of 63.1–154.7 mg/kg b.w. under low-level and moderate exposure to Cd is effective in counteracting numerous effects of this xenobiotic toxicity, including its disrupting impact on the homeostasis of Ca and P_i_ and the metabolism and properties of bone. Taking into account the available data in the literature on the average consumption of polyphenolic compounds (1000 mg/day [40]; i.e., 14.29 mg/kg b.w. [21]), we have estimated that a protective effect was noted at a polyphenol intake in female rats 2.9–7.3 times exceeding these compounds’ intake in humans. However, due to a lack of data on the average chokeberry consumption in the general population, a comparison of the effective extract dosage in animals with the consumption of aronia-based products in humans is difficult. The rationale may be the slightly tart and sour taste of chokeberries, with a noticeable hint of bitterness, which is why raw chokeberries are eaten reluctantly. However, *A. melanocarpa* berries are available as various processed products, mainly juices and extracts. An overview of data in the literature shows that daily consumption of about 100–300 mg of chokeberry extract or 150–300 mL of juice for a few to a dozen weeks, and sometimes higher doses (even 6 g) or for a longer time, was effective in numerous intervention studies to improve various parameters in healthy individuals or the health status in patients suffering from different diseases [68,69,70,71,72].

Although it is difficult to explain which components of the chokeberry extract were responsible for its protective impact and extrapolate AME intake in female rats to the consumption of chokeberry products in humans, this effect has been demonstrated and cannot be questioned. We are aware that apart from the novelty and important practical implications, our research also has some limitations. The first is conducting the study in female rats being more susceptible to Cd toxicity than males, and thus the possibility of extrapolating the finding from animals only to women. The next limitation is the inability to determine a larger number of variables involved in the maintenance of the homeostasis of Ca and P_i_ and regulating bone metabolism. This resulted from the limited amount of biological material, especially serum, that could be obtained from rats and a wide range of already performed measurements (the results of which are successively published by our research team). Moreover, we cannot explain some of our results, especially the unexpected decrease in 1,25(OH)_2_D_3_ concentration in the kidney of the females co-administered the diet with Cd at 1 mg/kg and AME for 17 months. However, despite these limitations, the results allowed us to draw clear conclusions. We are aware that our research should be continued and expanded before undertaking studies in this area in humans.

## 5. Conclusions

The results presented in this article, together with findings of our previous research in these animals, showed that low-to-moderate long-term exposure to Cd may exert an unfavourable impact on bone by influencing the pathways involved in regulating Ca and P_i_ metabolism and destroying the body status of these minerals. It seems that the possible pathways of the previously reported beneficial AME impact on the skeleton during exposure to this trace heavy metal involve the normalisation of serum concentrations of calciotropic hormones and Klotho and the improvement of the homeostasis of Ca and P_i_. The demonstration that the up-to-24-month supplementation with AME of animals fed a standard diet that contained Cd in only trace unavoidable amounts did not affect any of the determined indices of these bioelements’ metabolism and the pathways involved in maintaining their homeostasis shows that long-term consumption of chokeberry products under normal conditions does not disturb these minerals’ metabolism, whereas, in the case of exposure to a factor disturbing this metabolism like Cd, they will provide significant protection. Aronia products are widely known for their numerous health-promoting effects, including mainly antioxidative properties resulting from the high content of anthocyanins. The outcomes of the current study provided further evidence that supplementation with chokeberry products may be an important strategy to minimise the effects of environmental exposure to Cd and perhaps also to other factors that may disrupt the mineral balance and thus adversely affect the skeletal system. Considering the difficulty of avoiding environmental Cd exposure and the existence of many factors disrupting the mineral balance in the body, interest should be focused on the possibility of using chokeberry products as a potential strategy to prevent not only the outcomes of exposure to this toxic heavy metal but also bone diseases of other aetiologies.

## Figures and Tables

**Table 1:** Analytical quality of calcium (Ca) measurements in certified reference materials.

Certified Reference Material	Reference Values	Noticed Values ^1^	Recovery	Precision (CV) ^2^
Trace Elements Serum L-1 LOT 0903106 (SERO AS, Billingstad, Norway)	88.3–103.7 mg/L (mean 96.0 ± 7.7 mg/L)	92.8 ± 2.7 mg/L	97%	2.9%
Trace Elements Urine L-2 LOT 1011645 (Seronorm^TM^, Billingstad, Norway)	71 mg/L	67.3 ± 1.4 mg/L	95%	2.1%

^1^ Data are represented as mean ± standard error (SE) for three measurements. ^2^ The precision of measurements is expressed as a coefficient of variation (CV).

**Table 2:** Relationships between the determined indices of the metabolism of calcium (Ca) and inorganic phosphorus (P_i_) and markers of the pathways of its regulation in the female rats supplemented or not with 0.1% *Aronia melanocarpa* L. berry extract (AME) ^1^.

Parameter		Serum						Urine				Kidney	
		Ca	P_i_	1,25(OH)_2_D_3_	CT	PTH	Klotho	TE–Ca	FE–Ca	TE–P_i_	FE–P_i_	1alpha-OHase	1,25(OH)_2_D_3_
Serum	Ca		*0.206* ^a^	*0.233* ^a^	*NS*	*NS*	*NS*	*0.430* ^c^	*NS*	*NS*	*NS*	*NS*	−*0.474* ^c^
	P_i_	0.210 ^a^		*NS*	*NS*	*NS*	*NS*	*0.368* ^c^	*0.412* ^c^	*NS*	*NS*	*NS*	−*0.447* ^c^
	1,25(OH)_2_D_3_	−0.239 ^a^	0.261^a^		*0.203* ^a^	*NS*	*NS*	*0.233* ^a^	*NS*	*NS*	*NS*	*NS*	−*0.357* ^c^
	CT	−0.208 ^a^	NS	0.373 ^c^		*0.320* ^b^	NS	*NS*	−*0.232* ^a^	*NS*	*NS*	*0.227* ^a^	*NS*
	PTH	NS	NS	−0.413 ^c^	−0.352 ^c^		*NS*	*NS*	*NS*	*NS*	*NS*	*NS*	*NS*
	Klotho	−0.263 ^b^	NS	0.375 ^c^	0.600 ^c^	−0.476 ^c^		*NS*	*NS*	*NS*	*NS*	*NS*	*NS*
Urine	TE–Ca	0.549 ^c^	0.228 ^a^	NS	−0.364 ^c^	NS	−0.440 ^c^		*0.815* ^c^	*0.554* ^c^	*0.441* ^c^	*0.210* ^a^	−*0.610* ^c^
	FE–Ca	0.483 ^c^	NS	−0.225 ^a^	−0.423 ^c^	0.256 ^a^	−0.557 ^c^	0.891 ^c^		*0.287* ^b^	*0.385* ^c^	*NS*	−*0.439* ^c^
	TE–P_i_	0.261 ^b^	NS	NS	−0.346 ^c^	0.252 ^a^	NS	0.549 ^c^	0.468 ^c^		*0.818* ^c^	*NS*	−*0.212* ^a^
	FE–P_i_	0.370 ^c^	NS	−0.284 ^b^	−0.389 ^c^	0.300 ^b^	−0.366 ^c^	0.585 ^c^	0.699 ^c^	0.766 ^c^		*NS*	*NS*
Kidney	1alpha-OHase	NS	NS	0.287 ^b^	0.199 ^a^	−0.195 ^a^	0.192 ^c^	NS	NS	NS	−0.204 ^a^		*NS*
	1,25(OH)_2_D_3_	−0.501 ^c^	NS	0.438 ^c^	0.477 ^c^	−0.382 ^c^	0.708 ^c^	−0.575 ^c^	−0.670 ^c^	−0.305 ^b^	−0.492 ^c^	0.369 ^c^	

^1^ The results of Spearman’s correlation analysis are expressed as a correlation coefficient (r), the level of statistical significance (^a^ *p* < 0.05, ^b^ *p* < 0.01, and ^c^ *p* < 0.001), and NS for *p* > 0.05 (no correlation between two parameters). The italics values refer to the female rats supplied with AME (the AME group, Cd1 + AME group, and Cd5 + AME group). Other values refer to the females that received no AME (the control group, Cd1 group, and Cd5 group). CT, calcitonin; FE–Ca, fractional urinary excretion of calcium; FE–P_i_, fractional urinary excretion of inorganic phosphorus; PTH, parathormone; TE–Ca, total urinary excretion of calcium; TE–P_i_, total urinary excretion of inorganic phosphorus; 1alpha-OHase, 25-hydroxyvitamin 1alpha-hydroxylase; 1,25(OH)_2_D_3_, 1,25-dihydroxyvitamin D_3_.

**Table 3:** Relationships between the determined indices of the metabolism of calcium (Ca) and inorganic phosphorus (P_i_) and markers of the pathways of its regulation and cadmium (Cd) concentration in the body in animals with or without supplementation with 0.1% *Aronia melanocarpa* L. berry extract (AME) ^1,2^.

Parameter	Cd in the Blood		Cd in the Kidney		Cd in the Urine	
	Without AME	With AME	Without AME	With AME	Without AME	With AME
Serum Ca	NS	NS	NS	0.249 ^a^	NS	NS
TE–Ca	NS	NS	NS	0.319 ^b^	NS	NS
FE–Ca	0.202 ^a^	NS	0.357 ^c^	0.207 ^a^	NS	NS
Serum P_i_	−0.315 ^b^	NS	NS	NS	−0.272 ^b^	NS
TE–P_i_	NS	NS	0.251 ^a^	NS	0.246 ^a^	NS
FE–P_i_	0.378 ^c^	NS	0.482 ^c^	NS	0.397 ^c^	NS
Serum 1,25(OH)_2_D_3_	−0.556 ^c^	0.281 ^b^	−0.594 ^c^	0.280 ^b^	−0.406 ^c^	0.272 ^b^
Serum CT	−0.415 ^c^	NS	−0.472 ^c^	NS	−0.332 ^b^	NS
Serum PTH	0.459 ^c^	NS	0.535 ^c^	NS	0.417 ^c^	NS
Serum Klotho	−0.447 ^c^	NS	−0.548 ^c^	NS	−0.322 ^b^	NS
Kidney 1alpha-OHase	−0.475 ^c^	NS	−0.551 ^c^	NS	−0.548 ^c^	NS
Kidney 1,25(OH)_2_D_3_	−0.444 ^c^	−0.343 ^c^	−0.594 ^c^	−0.567 ^c^	−0.322 ^b^	−0.239 ^a^

^1^ The results of Spearman’s correlation analysis are expressed as a correlation coefficient (r), the level of statistical significance (^a^ *p* < 0.05, ^b^ *p* < 0.01, and ^c^ *p* < 0.001), and NS for *p* > 0.05 (no relationship between parameters) in female rats not supplemented with AME (without AME—the control group, Cd1 group, and Cd5 group) and those supplied with AME (the AME group, Cd1 + AME group, and Cd5 + AME group). ^2^ The concentration of Cd in the blood, kidney, and urine of these animals has already been reported [17]. CT, calcitonin; FE–Ca, fractional urinary excretion of calcium; FE–P_i_, fractional urinary excretion of inorganic phosphorus; TE–Ca, total urinary excretion of calcium; TE–P_i_, total urinary excretion of inorganic phosphorus; PTH, parathormone; 1alpha-OHase, 25-hydroxyvitamin D 1alpha-hydroxylase; 1,25(OH)_2_D_3_, 1,25-dihydroxyvitamin D_3_.

**Table 4:** Relationships between the determined indices of calcium (Ca) and inorganic phosphorus (P_i_) metabolism, markers of the pathways of its regulation, the markers of bone metabolism, and biomechanical properties in animals not supplemented with 0.1% *Aronia melanocarpa* L. berry extract (AME) ^1,2^.

Parameter		Serum						Urine				Kidney	
		Ca	P_i_	1,25(OH)_2_D_3_	CT	PTH	Klotho	TE–Ca	FE–Ca	TE–P_i_	FE–P_i_	1alpha-OHase	1,25(OH)_2_D_3_
Bone turnover													
Serum	OC	NS	NS	0.372 ^c^	NS	NS	NS	NS	NS	NS	NS	NS	0.257 ^a^
	ALP	0.317 ^b^	NS	NS	NS	NS	NS	0.357 ^c^	0.274 ^b^	NS	NS	NS	−0.221 ^a^
	CTX	−0.226 ^a^	NS	NS	NS	0.344 ^c^	−0.300 ^b^	NS	NS	NS	NS	NS	NS
Bone tissue—distal femoral epiphysis	ALP	−0.674 ^c^	NS	0.383 ^c^	0.540 ^c^	−0.309 ^b^	0.690 ^c^	−0.677 ^c^	−0.719 ^c^	−0.298 ^b^	−0.496 ^c^	NS	0.778 ^c^
	PC I	0.445 ^c^	0.253 ^a^	NS	NS	NS	−0.233 ^a^	0.408 ^c^	0.413 ^c^	NS	NS	NS	−0.369 ^c^
	OPG	−0.242 ^a^	0.271^b^	0.445 ^c^	0.362 ^c^	−0.357 ^c^	0.317 ^b^	NS	NS	−0.218 ^a^	−0.372 ^c^	0.347 ^c^	0.467 ^c^
	sRANKL	NS	NS	−0.252 ^a^	−0.460 ^c^	0.489 ^c^	−0.621 ^c^	0.292 ^b^	0.407 ^c^	0.204 ^a^	0.251^a^	NS	−0.409 ^c^
	sRANKL/OPG	NS	NS	−0.544 ^c^	−0.466 ^c^	0.605 ^c^	−0.598 ^c^	0.222 ^a^	0.353 ^c^	0.255 ^a^	0.398^c^	−0.360 ^c^	−0.635 ^c^
Mineral status													
Femur	% mineral comp. ^3^	−0.398 ^c^	NS	NS	0.380 ^c^	−0.307 ^b^	0.477 ^c^	−0.293 ^b^	−0.385 ^c^	NS	−0.354 ^c^	0.363 ^c^	0.615 ^c^
	Ca ^3^	−0.312 ^b^	NS	NS	0.424 ^c^	−0.306 ^b^	0.390 ^c^	−0.273 ^b^	−0.284 ^b^	−0.304 ^b^	−0.362 ^c^	0.272 ^b^	0.398 ^c^
	BMD	NS	0.308 ^b^	0.484 ^c^	0.218 ^a^	−0.229 ^a^	NS	0.275 ^b^	NS	NS	NS	0.279 ^b^	NS
Bone biomechanical properties													
Femoral neck	Yield strength	NS	NS	0.316 ^a^	NS	−0.266 ^b^	NS	NS	NS	−0.255 ^a^	NS	NS	NS
	Fracture strength	NS	NS	NS	NS	NS	NS	NS	NS	NS	NS	NS	NS
Femoral diaphysis	Yield strength	0.538 ^c^	0.278 ^b^	NS	NS	NS	−0.282 ^b^	0.510 ^c^	0.487 ^c^	NS	0.239 ^a^	NS	−0.427 ^c^
	Fracture strength	0.608 ^c^	0.242 ^a^	−0.249 ^a^	−0.306 ^b^	NS	−0.514 ^c^	0.590 ^c^	0.638 ^c^	NS	0.403 ^c^	NS	−0.670 ^c^

^1^ The results of Spearman’s correlation analysis are expressed as a correlation coefficient (r), the level of statistical significance (^a^ *p* < 0.05, ^b^ *p* < 0.01, and ^c^ *p* < 0.001), and NS for *p* > 0.05 (no relationship between parameters) in the female rats not supplemented with AME (the control group, Cd1 group, and Cd5 group). ^2^ The markers of bone metabolism and biomechanical properties have already been published [18,19]. ^3^ Percentage content of mineral components (% mineral comp.) and Ca concentration in the bone tissue at the distal femoral epiphysis [18]. ALP, alkaline phosphatase; BMD, bone mineral density; CT, calcitonin; CTX, carboxy-terminal cross-linking telopeptides of type I collagen; FE–Ca, fractional urinary excretion of calcium; FE–P_i_, fractional urinary excretion of inorganic phosphorus; OC, osteocalcin; OPG, osteoprotegerin; PC I, procollagen I; PTH, parathormone; sRANKL, soluble receptor activator of nuclear factor-κB ligand; sRANKL/OPG, the ratio of sRANKL and OPG; TE–Ca, total urinary excretion of calcium; TE–P_i_, total urinary excretion of inorganic phosphorus; 1alpha-OHase, 25-hydroxyvitamin D 1alpha-hydroxylase; 1,25(OH)_2_D_3_, 1,25-dihydroxyvitamin D_3_.

**Table 5:** Relationships between the determined indices of calcium (Ca) and inorganic phosphorus (P_i_) metabolism, pathways of its regulation, the markers of bone metabolism, and biomechanical properties in animals supplemented with 0.1% aqueous extract from *Aronia melanocarpa* L. berry extract (AME) ^1,2^.

Parameter		Serum						Urine				Kidney	
		Ca	P_i_	1,25(OH)_2_D_3_	CT	PTH	Klotho	TE–Ca	FE–Ca	TE–P_i_	FE–P_i_	1alpha-OHase	1,25(OH)_2_D_3_
Bone turnover													
Serum	OC	NS	NS	NS	NS	NS	NS	0.253 ^a^	NS	NS	NS	0.202 ^a^	−0.276 ^b^
	ALP	0.342 ^c^	0.321 ^b^	0.222 ^a^	−0.230 ^a^	−0.332 ^b^	NS	0.374 ^c^	0.250 ^a^	NS	NS	NS	−0.572 ^c^
	CTX	−0.369 ^c^	−0.252 ^a^	NS	NS	NS	NS	NS	−0.251 ^a^	NS	NS	NS	NS
Bone tissue—distal femoral epiphysis	ALP	−0.565 ^c^	−0.434 ^c^	−0.268 ^b^	0.216 ^a^	0.236 ^a^	NS	−0.552 ^c^	−0.486 ^c^	NS	NS	−0.321 ^b^	0.620 ^c^
	PC I	0.360 ^c^	0.319 ^b^	NS	NS	NS	NS	0.437 ^c^	0.412 ^c^	NS	NS	NS	−0.460 ^c^
	OPG	−0.311 ^b^	0.529 ^c^	0.250 ^a^	NS	NS	NS	0.438 ^c^	0.378 ^c^	NS	NS	0.341 ^c^	−0.664 ^c^
	sRANKL	−0.359 ^c^	NS	NS	NS	NS	−0.241 ^a^	NS	NS	NS	NS	NS	NS
	sRANKL/OPG	−0.491 ^c^	−0.447 ^c^	−0.218 ^a^	NS	NS	NS	−0.451 ^c^	−0.337 ^c^	NS	NS	−0.214 ^a^	0.618 ^c^
Mineral status													
Femur	% mineral comp. ^3^	−0.397 ^c^	−0.236 ^a^	−0.228 ^a^	NS	0.312 ^b^	NS	−0.356 ^c^	−0.252 ^a^	NS	NS	NS	0.660 ^c^
	Ca ^3^	−0.262 ^a^	NS	−0.230 ^a^	NS	NS	NS	NS	NS	NS	NS	NS	NS
	BMD	NS	0.300 ^b^	NS	NS	−0.272 ^b^	NS	0.326 ^b^	0.320 ^b^	NS	NS	0.246 ^a^	−0.331 ^b^
Bone biomechanical properties													
Femoral neck	Yield strength	NS	NS	NS	NS	NS	NS	NS	NS	NS	NS	NS	NS
	Fracture strength	NS	NS	NS	NS	NS	NS	NS	NS	NS	NS	NS	NS
Femoral diaphysis	Yield strength	0.468 ^c^	0.476 ^c^	0.301 ^b^	NS	NS	NS	0.605 ^c^	0.475 ^c^	NS	NS	0.273 ^b^	−0.733 ^c^
	Fracture strength	0.433 ^c^	0.495 ^c^	0.350 ^c^	NS	NS	NS	0.537 ^c^	0.417 ^c^	NS	NS	0.225 ^a^	−0.774 ^c^

^1^ The results of Spearman’s correlation analysis are expressed as a correlation coefficient (r), the level of statistical significance (^a^ *p* < 0.05, ^b^ *p* < 0.01, and ^c^ *p* < 0.001), and NS for *p* > 0.05 (no relationship between parameters) in the female rats supplemented with AME (the AME group, Cd1 + AME group, and Cd5 + AME group). ^2^ The markers of bone metabolism and biomechanical properties have already been published [18,19]. ^3^ Percentage content of mineral components (% mineral comp.) and Ca concentration in the bone tissue at the distal femoral epiphysis [18]. ALP, alkaline phosphatase; BMD, bone mineral density; CT, calcitonin; CTX, carboxy-terminal cross-linking telopeptides of type I collagen; FE–Ca, fractional urinary excretion of calcium; FE–P_i_, fractional urinary excretion of inorganic phosphorus; OC, osteocalcin; OPG, osteoprotegerin; PC I, procollagen I; PTH, parathormone; sRANKL, soluble receptor activator of nuclear factor-κB ligand; sRANKL/OPG, the ratio of sRANKL and OPG; TE–Ca, total urinary excretion of calcium; TE–P_i_, total urinary excretion of inorganic phosphorus; 1alpha-OHase, 25-hydroxyvitamin D 1alpha-hydroxylase; 1,25(OH)_2_D_3_, 1,25-dihydroxyvitamin D_3_.

**Table 6:** Relationships between the determined indices of calcium (Ca) and inorganic phosphorus (P_i_) metabolism, pathways of its regulation, and the markers of kidney function in animals not supplemented with 0.1% *Aronia melanocarpa* L. berry extract (AME) ^1,2^.

Parameter		Serum						Urine				Kidney	
		Ca	P_i_	1,25(OH)_2_D_3_	CT	PTH	Klotho	TE–Ca	FE–Ca	TE–P_i_	FE–P_i_	1alpha-OHase	1,25(OH)_2_D_3_
Urine	KIM-1	NS	NS	−0.433 ^c^	−0.361 ^c^	0.523 ^c^	−0.460 ^c^	NS	0.333 ^b^	0.244 ^a^	0.369 ^c^	−0.272 ^b^	−0.367 ^c^
	β2-MG	0.433 ^c^	NS	−0.438 ^c^	−0.592 ^c^	0.399 ^c^	−0.579 ^c^	0.581 ^c^	0.710 ^c^	0.453 ^c^	0.644 ^c^	−0.312 ^b^	−0.658 ^c^
	NAG	0.493 ^c^	NS	−0.548 ^c^	−0.452 ^c^	0.416 ^c^	−0.451 ^c^	0.392 ^c^	0.489 ^c^	0.218 ^a^	0.471 ^c^	−0.320 ^b^	−0.608 ^c^
	ACR	0.359 ^c^	NS	−0.420 ^c^	−0.564 ^c^	0.467 ^c^	−0.608 ^c^	0.531 ^c^	0.688 ^c^	0.530 ^c^	0.728 ^c^	−0.256 ^a^	−0.622 ^c^
	PCR	0.514 ^c^	NS	−0.520 ^c^	−0.469 ^c^	0.392 ^c^	−0.540 ^c^	0.419 ^c^	0.561 ^c^	0.218 ^a^	0.500 ^c^	−0.324 ^b^	−0.677 ^c^
	ALP	0.420 ^c^	NS	−0.623 ^c^	−0.342 ^c^	0.356 ^c^	−0.385 ^c^	NS	0.294 ^b^	NS	0.309 ^b^	−0.313 ^b^	−0.493 ^c^
	Uric acid	NS	NS	NS	NS	NS	NS	0.212 ^a^	NS	0.686 ^c^	0.494 ^c^	NS	NS
	Urea	NS	NS	NS	−0.232 ^a^	0.221 ^a^	NS	0.340 ^c^	0.309 ^b^	0.602 ^c^	0.425 ^c^	NS	NS
Serum	Uric acid	NS	NS	NS	−0.225 ^a^	0.253 ^a^	−0.454 ^c^	0.287 ^b^	0.319 ^b^	NS	NS	NS	−0.278 ^b^
	Urea	NS	−0.220 ^a^	−0.289 ^b^	−0.233 ^a^	0.301 ^b^	−0.221 ^a^	NS	0.329 ^b^	0.210 ^a^	0.402 ^c^	−0.203 ^a^	−0.338 ^c^
Creatinine clearance		−0.288 ^b^	NS	0.294 ^b^	0.225 ^a^	NS	0.475 ^c^	−0.266 ^b^	−0.577 ^c^	NS	−0.495 ^c^	NS	0.519 ^c^

^1^ The results of Spearman’s correlation analysis are expressed as a correlation coefficient (r), the level of statistical significance (^a^ *p* < 0.05, ^b^ *p* < 0.01, and ^c^ *p* < 0.001), and NS for *p* > 0.05 (no relationship between parameters) in female rats not supplemented with AME (the control group, Cd1 group, and Cd5 group). ^2^ The markers of kidney function have already been published [21]. ACR, albumin concentration in the urine (expressed in calculation per creatinine); ALP, alkaline phosphatase; CT, calcitonin; FE–Ca, fractional urinary excretion of calcium; FE–P_i_, fractional urinary excretion of inorganic phosphorus; KIM-1, kidney injury molecule-1; NAG, *N*-acetyl-β-D-glucosaminidase; PCR, total protein concentration in the urine (expressed in calculation per creatinine); PTH, parathormone; TE–Ca, total urinary excretion of calcium; TE–P_i_, total urinary excretion of inorganic phosphorus; β2-MG, β2-microglobulin; 1alpha-OHase, 25-hydroxyvitamin D 1alpha-hydroxylase; 1,25(OH)_2_D_3_, 1,25-dihydroxyvitamin D_3_.

**Table 7:** Relationships between the determined indices of calcium (Ca) and inorganic phosphorus (P_i_) metabolism, pathways of its regulation, the markers of bone metabolism, and biomechanical properties in animals supplemented with 0.1% *Aronia melanocarpa* L. berry extract (AME) ^1,2^.

Parameter		Serum						Urine				Kidney	
		Ca	P_i_	1,25(OH)_2_D_3_	CT	PTH	Klotho	TE–Ca	FE–Ca	TE–P_i_	FE–P_i_	1alpha-OHase	1,25(OH)_2_D_3_
Urine	KIM-1	NS	0.365 ^c^	NS	NS	NS	NS	0.373 ^c^	0.325 ^b^	0.375 ^c^	0.281 ^b^	0.308 ^b^	−0.321 ^b^
	β2-MG	0.300 ^b^	0.453 ^c^	NS	NS	NS	NS	0.524 ^c^	0.472 ^c^	0.437 ^c^	0.417 ^c^	0.223 ^a^	−0.487 ^c^
	NAG	0.237 ^a^	0.313 ^b^	NS	NS	NS	NS	0.251 ^a^	0.212 ^a^	NS	NS	NS	−0.389 ^c^
	ACR	0.356 ^c^	0.416 ^c^	0.229 ^a^	NS	NS	NS	0.388 ^c^	0.317 ^b^	0.272 ^b^	0.226 ^a^	0.286 ^b^	−0.483 ^c^
	PCR	0.232 ^a^	0.211 ^a^	NS	NS	NS	NS	NS	0.247 ^a^	NS	NS	NS	−0.305 ^b^
	ALP	0.346 ^c^	NS	NS	NS	NS	NS	NS	NS	NS	NS	NS	−0.277 ^b^
	Uric acid	NS	NS	NS	NS	NS	NS	0.378 ^c^	0.262 ^a^	0.611 ^c^	0.579 ^c^	NS	NS
	Urea	NS	NS	NS	NS	NS	NS	NS	NS	0.554 ^c^	0.470 ^c^	NS	NS
Serum	Uric acid	NS	NS	0.262 ^b^	NS	NS	NS	NS	NS	NS	NS	0.436 ^c^	−0.245 ^a^
	Urea	NS	NS	−0.250 ^a^	NS	NS	NS	0.226 ^a^	NS	NS	NS	NS	NS
Creatinine clearance		NS	NS	NS	NS	NS	NS	NS	−0.349 ^c^	0.338 ^c^	NS	NS	NS

^1^ The results of Spearman’s correlation analysis are expressed as a correlation coefficient (r), the level of statistical significance (^a^ *p* < 0.05, ^b^ *p* < 0.01, and ^c^ *p* < 0.001), and NS for *p* > 0.05 (no relationship between parameters) in the female rats supplemented with AME (the AME group, Cd1 + AME group, and Cd5 + AME group). ^2^ The markers of kidney function have already been published [21]. ACR, albumin concentration in the urine (expressed in calculation per creatinine); ALP, alkaline phosphatase; CT, calcitonin; FE–Ca, fractional urinary excretion of calcium; FE–P_i_, fractional urinary excretion of inorganic phosphorus; KIM-1, kidney injury molecule-1; NAG, *N*-acetyl-β-D-glucosaminidase; PCR, total protein concentration in the urine (expressed in calculation per creatinine); PTH, parathormone; TE–Ca, total urinary excretion of calcium; TE–P_i_, total urinary excretion of inorganic phosphorus; β2-MG, β2-microglobulin; 1alpha-OHase, 25-hydroxyvitamin D 1alpha-hydroxylase; 1,25(OH)_2_D_3_, 1,25-dihydroxyvitamin D_3_.

## Data Availability

The data presented in this study are available on request from the corresponding authors. The data are not publicly available.

## Supplementary Materials

The following supporting information can be downloaded at: https://www.mdpi.com/article/10.3390/nu17040702/s1, Table S1: Exposure to cadmium (Cd) and/or supplementation with an extract from the berries of *Aronia melanocarpa* L. (AME) impact on the metabolism and bone biomechanical properties in female rats; Table S2: Exposure to cadmium (Cd) and/or supplementation with an extract from the berries of *Aronia melanocarpa* L. (AME) impact on the biomarkers of kidney damage in female rats; Table S3: Exposure to cadmium (Cd) and/or supplementation with an extract from the berries of *Aronia melanocarpa* L. (AME) impact on the concentration of calcium (Ca) in the serum and its urinary total excretion (TE–Ca) and fractional excretion (FE–Ca) in female rats; Table S4: Exposure to cadmium (Cd) and/or supplementation with an extract from the berries of *Aronia melanocarpa* L. (AME) impact on the concentration of inorganic phosphorus (P_i_) in the serum and its urinary total excretion (TE–P_i_) and fractional excretion (FE–P_i_) in female rats; Table S5: Exposure to cadmium (Cd) and/or supplementation with an extract from the berries of *Aronia melanocarpa* L. (AME) impact on the concentrations of 1,25-dihydroxyvitamin D_3_ (1,25(OH)_2_D_3_), calcitonin (CT), and parathormone (PTH) in the serum of female rats; Table S6: Exposure to cadmium (Cd) and/or supplementation with an extract from the berries of *Aronia melanocarpa* L. (AME) impact on the concentration of Klotho in the serum of female rats; Table S7: Exposure to cadmium (Cd) and/or supplementation with an extract from the berries of *Aronia melanocarpa* L. (AME) impact on the concentrations of 1,25-dihydroxyvitamin D_3_ (1,25(OH)_2_D_3_) and 25-hydroxyvitamin D 1alpha-hydroxylase (1alpha-OHase) in the kidney of female rats.

## Abbreviations

AAS method, atomic absorption spectrometry; ACR, albumin concentration in the urine adjusted for creatinine concentration; ALP, alkaline phosphatase; AME, an aqueous extract from *Aronia melanocarpa* L. (Michx.) Elliott berries (chokeberries); AMP, *Aronia melanocarpa* L. polyphenols; BMD, bone mineral density; Ca, calcium; Ca^2+^, calcium ion; Ca-BP, calcium-binding protein; Cd, cadmium; CdCl_2_ × 2.5 H_2_O_2_, hydrated cadmium chloride; Cd1 group, a group of females exposed to cadmium in a diet at the concentration of 1 mg/kg; Cd1 + AME group, a group of females supplemented with 0.1% aqueous extract from *A. melanocarpa* L. berries during the exposure to cadmium at 1 mg/kg of diet; Cd5 group, a group of females exposed to cadmium in a diet at the concentration of 5 mg/kg; Cd5 + AME group, a group of females supplemented with 0.1% aqueous extract from *A. melanocarpa* L. berries during the exposure to cadmium at 5 mg/kg of diet; Cd^2+^, cadmium ion; CT, calcitonin; CTX, carboxy-terminal cross-linking telopeptides of type I collagen; CV, coefficient of variation; ELISA, enzyme-linked immunosorbent assay; FE, fractional excretion with urine; FE–Ca, fractional urinary excretion of calcium; FE–P_i_, fractional urinary excretion of inorganic phosphorus; FGF-23, fibroblast growth factor 23; GSH, reduced glutathione; KIM-1, kidney injury molecule-1; NAG, *N*-acetyl-β-D-glucosaminidase; OC, osteocalcin; OPG, osteoprotegerin; PC I, procollagen I; PCR, total protein concentration in the urine adjusted for creatinine concentration; P_i_, inorganic phosphorus; PTH, parathormone; SD, standard deviation; SE, standard error; sRANKL, soluble receptor activator of nuclear factor-κB ligand; TE–Ca, total urinary excretion of calcium; TE–P_i_, total urinary excretion of inorganic phosphorus; β2-MG, β2-microglobulin; η^2^, eta squared (the effect size is defined as the strength of the difference between the tested groups); 1alpha-OHase, 25-hydroxyvitamin D 1alpha-hydroxylase; 25(OH)D_3_, 25-hydroksyvitamin D_3_; 1,25(OH)_2_D_3_, 1,25-dihydroxyvitamin D_3_.

## References

[1] Clynes M.A., Harvey N.C., Curtis E.M., Fuggle N.R., Dennison E.M., Cooper C. (2020). The epidemiology of osteoporosis. Br. Med. Bull..

[2] Khandelwal S., Lane N.E. (2023). Osteoporosis. Review of etiology, mechanisms, and approach to management in the aging population. Endocrinol. Metab. Clin. N. Am..

[3] Tański W., Kosiorowska J., Szymańska-Chabowska A. (2021). Osteoporosis—Risk factors, pharmaceutical and non-pharmaceutical treatment. Eur. Rev. Med. Pharmacol. Sci..

[4] Kunioka C.T., Manso M.C., Carvalho M. (2023). Association between environmental cadmium exposure and osteoporosis risk in postmenopausal women: A systematic review and meta-analysis. Int. J. Environ. Res. Public Health.

[5] Buha A., Jugdaohsingh R., Matovica V., Bulata Z., Antonijevica B., Kernsc J.G., Goodshipd A., Hartd A., Powell J.J. (2019). Bone mineral health is sensitively related to environmental cadmium exposure—Experimental and human data. Environ. Res..

[6] Malin Igra A., Vahter M., Raqib R., Kippler M. (2019). Early-life cadmium exposure and bone-related biomarkers: A longitudinal study in children. Environ. Health Perspect..

[7] Huang X., Liu T., Zhao M., Fu H., Wang J., Xu Q. (2019). Protective effects of moderate Ca supplementation against Cd-induced bone damage under different population-relevant doses in young female rats. Nutrients.

[8] Lei Y., Guo M., Xie J., Liu X., Li X., Wang H., Xu Y., Zheng D. (2024). Relationship between blood cadmium levels and bone mineral density in adults: A cross-sectional study. Front. Endocrinol..

[9] Cirovic A., Cirovic A., Yimthiang S., Vesey D.A., Satarug S. (2024). Modulation of adverse health effects of environmental cadmium exposure by zinc and its transporters. Biomolecules.

[10] Schaefer H.R., Dennis S., Fitzpatrick S. (2020). Cadmium: Mitigation strategies to reduce dietary exposure. J. Food Sci..

[11] Satarug S. (2024). Is chronic kidney disease due to cadmium exposure inevitable and can it be reversed?. Biomedicines.

[12] Ishibashi Y., Harada S., Eitaki Y., Kurihara A., Kato S., Kuwabara K., Iida M., Hirata A., Sata M., Matsumoto M. (2024). A population-based urinary and plasma metabolomics study of environmental exposure to cadmium. Environ. Health Prev. Med..

[13] Verzelloni P., Urbano T., Wise L.A., Vinceti M., Filippini T. (2024). Cadmium exposure and cardiovascular disease risk: A systematic review and dose-response meta-analysis. Environ. Pollut..

[14] Arruebarrena M.A., Hawe C.T., Lee Y.M., Branco R.C. (2023). Mechanisms of cadmium neurotoxicity. Int. J. Mol. Sci..

[15] Cui Z.G., Ahmed K., Zaidi S.F., Muhammad J.S. (2021). Ins and outs of cadmium-induced carcinogenesis: Mechanism and prevention. Cancer Treat. Res. Commun..

[16] Xiao L., Li W., Zhu C., Yang S., Zhou M., Wang B., Wang X., Wang D., Ma J., Zhou Y. (2021). Cadmium exposure, fasting blood glucose changes, and type 2 diabetes mellitus: A longitudinal prospective study in China. Environ. Res..

[17] Brzóska M.M., Gałażyn-Sidorczuk M., Jurczuk M., Tomczyk M. (2015). Protective effect of *Aronia melanocarpa* polyphenols on cadmium accumulation in the body: A study in a rat model of human exposure to this metal. Curr. Drug Targets.

[18] Brzóska M.M., Rogalska J., Gałażyn-Sidorczuk M., Jurczuk M., Roszczenko A., Tomczyk M. (2015). Protective effect of *Aronia melanocarpa* polyphenols against cadmium-induced disorders in bone metabolism: A study in a rat model of lifetime human exposure to this heavy metal. Chem. Biol. Interact..

[19] Brzóska M.M., Roszczenko A., Rogalska J., Gałażyn-Sidorczuk M., Mężyńska M. (2017). Protective effect of chokeberry (*Aronia melanocarpa* L.) extract against cadmium impact on the biomechanical properties of the femur: A study in a rat model of low and moderate lifetime women exposure to this heavy metal. Nutrients.

[20] Brzóska M.M., Rogalska J., Roszczenko A., Gałażyn-Sidorczuk M., Tomczyk M. (2016). The mechanism of the osteoprotective action of a polyphenol-rich *Aronia melanocarpa* extract during chronic exposure to cadmium is mediated by the oxidative defense system. Planta Med..

[21] Smereczański N.M., Brzóska M.M., Rogalska J., Hutsch T. (2023). The protective potential of *Aronia melanocarpa* L. berry extract against cadmium-induced kidney damage: A study in an animal model of human environmental exposure to this toxic element. Int. J. Mol. Sci..

[22] Brzóska M.M., Gałażyn-Sidorczuk M., Kozłowska M., Smereczański N.M. (2022). The body status of manganese and activity of this element-dependent mitochondrial superoxide dismutase in a rat model of human exposure to cadmium and co-administration of *Aronia melanocarpa* L. extract. Nutrients.

[23] Ruczaj A., Brzóska M.M., Rogalska J. (2024). The protective impact of *Aronia melanocarpa* L. berries extract against prooxidative cadmium action in the brain—A study in an in vivo model of current environmental human exposure to this harmful element. Nutrients.

[24] Hashim M., Arif H., Tabassum B., Rehman S., Bajaj P., Sirohi R., Khan M.F.A. (2024). An overview of the ameliorative efficacy of *Catharanthus roseus* extract against Cd^2+^ toxicity: Implications for human health and remediation strategies. Front. Public Health.

[25] Zwolak I. (2020). The role of selenium in arsenic and cadmium toxicity: An updated review of scientific literature. Biol. Trace Elem. Res..

[26] Mognetti B., Franco F., Castrignano C.H., Bovolin P., Berta G.N. (2024). Mechanisms of phytoremediation by resveratrol against cadmium toxicity. Antioxidants.

[27] Sandbichler A.M., Höckner M. (2016). Cadmium protection strategies—A hidden trade-off?. Int. J. Mol. Sci..

[28] Markiewicz-Górka I. (2019). Alleviating effect of α-lipoic acid and magnesium on cadmium-induced inflammatory processes, oxidative stress and bone metabolism disorders in Wistar rats. Int. J. Environ. Res. Public Health.

[29] Brzóska M.M., Moniuszko-Jakoniuk J. (2004). Low-level exposure to cadmium during the lifetime increases the risk of osteoporosis and fractures of the lumbar spine in the elderly: Studies on a rat model of human environmental exposure. Toxicol. Sci..

[30] Brzóska M.M., Moniuszko-Jakoniuk J. (2005). Effect of exposure to cadmium on the mineral status and mechanical properties of lumbar spine of male rats. Toxicol. Lett..

[31] Brzóska M.M., Moniuszko-Jakoniuk J. (2005). Effect of low-level lifetime exposure to cadmium on calciotropic hormones in aged female rats. Arch. Toxicol..

[32] Murshed M. (2018). Mechanism of bone mineralization. Cold Spring Harb. Perspect. Med..

[33] Patergnani S., Danese A., Bouhamida E., Aguiari G., Previati M., Pinton P., Giorgi C. (2020). Various aspects of calcium signaling in the regulation of apoptosis, autophagy, cell proliferation, and cancer. Int. J. Mol. Sci..

[34] Jacquillet G., Unwin R.J. (2019). Physiological regulation of phosphate by vitamin D, parathyroid hormone (PTH) and phosphate (Pi). Pflugers Arch. Eur. J. Physiol..

[35] Chacar F.C., Kogika M.M., Zafalon R.V.A., Brunetto M.A. (2020). Vitamin D metabolism and its role in mineral and bone disorders in chronic kidney disease in humans, dogs and cats. Metabolites.

[36] Chwalba A., Orłowska J., Słota M., Jeziorska M., Filipecka K., Bellanti F., Dobrakowski M., Kasperczyk A., Zalejska-Fiolka J., Kasperczyk S. (2023). Effect of cadmium on oxidative stress indices and vitamin D concentrations in children. J. Clin. Med..

[37] Babić Leko M., Pleić N., Gunjača I., Zemunik T. (2022). Environmental factors that affect parathyroid hormone and calcitonin levels. Int. J. Mol. Sci..

[38] Andreollo N.A., Santos E.F., Araújo M.R., Lopes L.R. (2012). Rat’s age versus human’s age: What is the relationship?. Arq. Bras. Cir. Dig..

[39] Sengupta P. (2013). The laboratory rat: Relating its age with human’s. Int. J. Prev. Med..

[40] Del Bo’ C., Bernardi S., Marino M., Porrini M., Tucci M., Guglielmetti S., Cherubini A., Carrieri B., Kirkup B., Kroon P. (2019). Systematic review on polyphenol intake and health outcomes: Is there sufficient evidence to define a health-promoting polyphenol-rich dietary pattern?. Nutrients.

[41] Murray S.L., Wolf M. (2024). Calcium and phosphate disorders: Core curriculum 2024. Am. J. Kidney Dis..

[42] Bian A., Xing C., Hu M.C. (2014). Alpha Klotho and phosphate homeostasis. J. Endocrinol. Investig..

[43] Tan S.J., Smith E.R., Holt S.G., Hewitson T.D., Toussaint N.D. (2017). Soluble Klotho may be a marker of phosphate reabsorption. Clin. Kidney J..

[44] Huang C.L., Moe O.W. (2011). Klotho: A novel regulator of calcium and phosphorus homeostasis. Flugers Arch. Eur. J. Physiol..

[45] Toro-Román V., Robles-Gil M.C., Munoz D., Bartolomé I., Grijota F.J., Maynar-Marino M. (2023). Sex differences in cadmium and lead concentrations in different biological matrices in athletes. Relationship with iron status. Environ. Toxicol. Pharmacol..

[46] Lee B.K., Kim Y. (2014). Sex-specific profiles of blood metal levels associated with metal–iron interactions. Saf. Health Work.

[47] Sidor A., Gramza-Michałowska A. (2019). Black chokeberry *Aronia melanocarpa* L.—A qualitative composition, phenolic profile and antioxidant potential. Molecules.

[48] Han J., Wu L., Lv X., Liu M., Zhang Y., He L., Hao J., Xi L., Qu H., Shi C. (2023). Intestinal segment and vitamin D3 concentration affect gene expression levels of calcium and phosphorus transporters in broiler chickens. J. Anim. Sci. Technol..

[49] Zhu M., Zhou W., Bai L., Li H., Wang L., Zou X. (2019). Dietary cadmium chloride supplementation impairs renal function and bone metabolism in laying hens. Animals.

[50] Yuan G., Lu H., Yin Z., Dai S., Jia R., Xu J., Song X., Li L. (2014). Effects of mixed subchronic lead acetate and cadmium chloride on bone metabolism in rats. Int. J. Clin. Exp. Med..

[51] Delrue C., Speeckaert M.M. (2023). Vitamin D and vitamin D-binding protein in health and disease. Int. J. Mol. Sci..

[52] Engström A., Skerving S., Lidfeldt J., Burgaz A., Lundh T., Samsioe G., Vahter M., Åkesson A. (2009). Cadmium-induced bone effect is not mediated via low serum 1,25-dihydroxy vitamin D. Environ. Res..

[53] Chen X., Dai Y., Wang Z., Zhu G., Ding X., Jin T. (2018). The association between serum vitamin D levels and renal tubular dysfunction in a general population exposed to cadmium in China. PLoS ONE.

[54] Niwano Y., Kohzaki H., Shirato M., Shishido S., Nakamura K. (2022). Anti-osteoporotic mechanisms of polyphenols elucidated based on in vivo studies using ovariectomized animals. Antioxidants.

[55] Nicolin V., De Tommasi N., Nori S.L., Costantinides F., Berton F., Di Lenarda R. (2019). Modulatory effects of plant polyphenols on bone remodeling: A prospective view from the bench to bedside. Front. Endocrinol..

[56] Zheng Y., Wang J., Xu K., Chen X. (2024). Intake of dietary flavonoids in relation to bone loss among U.S. adults: A promising strategy for improving bone health. Food Funct..

[57] Martiniakova M., Babikova M., Mondockova V., Blahova J., Kovacova V., Omelka R. (2022). The role of macronutrients, micronutrients and flavonoid polyphenols in the prevention and treatment of osteoporosis. Nutrients.

[58] Hodges J.K., Maiz M., Cao S., Lachcik P.J., Peacock M., McCabe G.P., McCabe L.D., Cladis D.P., Jackson G.S., Ferruzzi M.G. (2023). Moderate consumption of freeze-dried blueberry powder increased net bone calcium retention compared with no treatment in healthy postmenopausal women: A randomized crossover trial. Am. J. Clin. Nutr..

[59] Domazetovic V., Marcucci G., Falsetti I., Bilia A.R., Vincenzini M.T., Brandi M.L., Iantomasi T. (2020). Blueberry juice antioxidants protect osteogenic activity against oxidative stress and improve long-term activation of the mineralization process in human osteoblast-like SaOS-2 cells: Involvement of SIRT1. Antioxidants.

[60] Faienza M.F., Giardinelli S., Annicchiarico A., Chiarito M., Barile B., Corbo F., Brunetti G. (2024). Nutraceuticals and functional foods: A comprehensive review of their role in bone health. Int. J. Mol. Sci..

[61] Hassan J.K., Sharrad A.K., Sheri F.H. (2018). Effect of quercetin supplement on some bone mineralization biomarkers in diabetic type 2 patients. Adv. Pharmacol. Pharm..

[62] Marchionatti A.M., Pacciaroni A., Tolosa de Talamoni N.G. (2013). Effects of quercetin and menadione on intestinal calcium absorption and the underlying mechanisms. Comp. Biochem. Physiol. A.

[63] Wong S.K., Chin K.Y., Ima-Nirwana S. (2020). Quercetin as an agent for protecting the bone: A review of the current evidence. Int. J. Mol. Sci..

[64] Abd El-Fattah A.I., Fathy M.M., Ali Z.Y., El-Garawany A.E.A., Mohamed E.K. (2017). Enhanced therapeutic benefit of quercetin-loaded phytosome nanoparticles in ovariectomized rats. Chem. Biol. Interact..

[65] Ho C.Y., Tang C.H., Ho T.L., Wang W.L., Yao C.H. (2024). Chlorogenic acid prevents ovariectomized-induced bone loss by facilitating osteoblast functions and suppressing osteoclast formation. Aging.

[66] Hu B., Chen L., Chen Y., Zhang Z., Wang X., Zhou B. (2021). Cyanidin-3-glucoside regulates osteoblast differentiation via the ERK1/2 signaling pathway. ACS Omega.

[67] Cladis D.P., Debelo H., Lachcik P.J., Ferruzzi M.G., Weaver C.M. (2020). Increasing doses of blueberry polyphenols alters colonic metabolism and calcium absorption in ovariectomized rats. Mol. Nutr. Food Res..

[68] Olechno E., Puścion-Jakubik A., Zujko M.E. (2022). Chokeberry (*A. melanocarpa* (Michx.) Elliott)—A natural product for metabolic disorders?. Nutrients.

[69] Chung J.W., Kim J.E., Nam Y.E., Kim W.S., Lee I., Yim S.V., Kwon O. (2023). Eight-week supplementation of *Aronia* berry extract promoted the glutathione defence system against acute aerobic exercise-induced oxidative load immediately and 30 min post-exercise in healthy adults: A double-blind, randomised controlled trial. J. Hum. Nutr. Diet.

[70] Milutinovic M., Velickovic Radovanovic R., Savikin K., Radenkovic S., Arvandi M., Pesic M., Kostic M., Miladinovic B., Brankovic S., Kitic D. (2019). Chokeberry juice supplementation in type 2 diabetic patients—Impact on health status. J. Appl. Biomed..

[71] Ahles S., Stevens Y.R., Joris P.J., Vauzour D., Adam J., de Groot E., Plat J. (2020). The effect of long-term *Aronia melanocarpa* extract supplementation on cognitive performance, mood, and vascular function: A randomized controlled trial in healthy, middle-aged individuals. Nutrients.

[72] Stankiewicz B., Cieślicka M., Mieszkowski J., Kochanowicz A., Niespodziński B., Szwarc A., Waldziński T., Reczkowicz J., Piskorska E., Petr M. (2023). Effect of supplementation with black chokeberry (*Aronia melanocarpa*) extract on inflammatory status and selected markers of iron metabolism in young football players: A randomized double-blind trial. Nutrients.

