# Supplementary material for: The Preventive Impact of Chokeberry (Aronia melanocarpa L.) Extract Regarding the Disruption of Calcium and Phosphorus Homeostasis and Chosen Pathways of Its Regulation in an Animal Model of General Population Exposure to Cadmium
Source: Nutrients. 2025 Feb 16;17(4):702. doi: 10.3390/nu17040702 (PMC11858106; doi:10.3390/nu17040702)
Supplement: Supplementary file 1 [file nutrients-17-00702-s001.zip › nutrients-3432042-supplementary.pdf]

nutrients

Supplementary Materials

The Preventive Impact of Chokeberry (*Aronia melanocarpa* L.) Extract Regarding the Disruption of Calcium and Phosphorus Homeostasis and Chosen Pathways of Its Regulation in an Animal Model of General Population Exposure to Cadmium

Małgorzata M. Brzóska *, Małgorzata Gałażyn-Sidorczuk and Joanna Rogalska

Department of Toxicology, Medical University of Bialystok, Adama Mickiewicza 2C Street, 15-222 Bialystok, Poland; malgorzata.galazyn-sidorczuk@umb.edu.pl (M.G.-S.); joanna.rogalska@umb.edu.pl (J.R.)

* Correspondence: malgorzata.brzoska@umb.edu.pl; Tel.: +48-85-748-5604; Fax: +48-85-748-5834

Table S1. Exposure to cadmium (Cd) and/or supplementation with an extract from the berries of *Aronia melanocarpa* L. (AME) impact on the metabolism and bone biomechanical properties in female rats. [1]

Parameters	Group	Experimental Duration							
		3 Months		10 Months		17 Months		24 Months	
**Mineral status**									
Femoral BMD	Cd1	↔		↓		↓		↓	
	Cd1 + AME	↑	↔	↔	↗	↔	↗	↔	↗
	Cd5	↔		↓		↓		↓	
	Cd5 + AME	↔	↔	↓	↗	↓	↗	↔	↗
% mineral components in the bone tissue at the distal femoral epiphysis	Cd1	↔		↓		↓		↓	
	Cd1 + AME	↔	↗	↓	↗	↔	↗	↔	↗
	Cd5	↓		↓		↓		↓	
	Cd5 + AME	↓	↗	↓	↗	↔	↗	↔	↗
Ca concentration in the bone tissue at the distal femoral epiphysis	Cd1	↔		↓		↓		↓	
	Cd1 + AME	↔	↔	↔	↗	↔	↗	↔	↗
	Cd5	↔		↓		↓		↓	
	Cd5 + AME	↔	↔	↔	↗	↔	↗	↔	↗
**Bone turnover**									
Serum OC	Cd1	↔		↓		↓		↔	
	Cd1 + AME	↔	↔	↔	↗	↔	↗	↔	↔
	Cd5	↔		↓		↓		↔	
	Cd5 + AME	↔	↔	↔	↗	↔	↗	↑	↗
Serum ALP	Cd1	↔		↔		↓		↓	
	Cd1 + AME	↔	↔	↔	↔	↔	↔	↔	↗
	Cd5	↔		↓		↓		↓	
	Cd5 + AME	↔	↔	↔	↗	↔	↔	↔	↗
ALP in the bone tissue at the distal femoral epiphysis	Cd1	↔		↓		↓		↓	
	Cd1 + AME	↔	↔	↓	↔	↔	↗	↑	↗
	Cd5	↔		↓		↓		↓	
	Cd5 + AME	↔	↔	↔	↗	↓	↗	↑	↗
PC I in the bone tissue at the distal femoral epiphysis	Cd1	↔		↓		↓		↓	
	Cd1 + AME	↓	↔	↔	↗	↓	↗	↔	↗
	Cd5	↓		↓		↓		↓	
	Cd5 + AME	↔	↗	↔	↗	↔	↗	↔	↗
PC I in the bone tissue at the femoral diaphysis	Cd1	↔		↓		↓		↔	
	Cd1 + AME	↔	↔	↓	↔	↓	↗	↔	↔
	Cd5	↔		↓		↓		↓	
	Cd5 + AME	↔	↗	↓	↔	↓	↗	↔	↗
OPG in the bone tissue at the distal femoral epiphysis	Cd1	↔		↓		↓		↓	
	Cd1 + AME	↔	↔	↔	↔	↑	↗	↑	↗
	Cd5	↔		↓		↓		↓	
	Cd5 + AME	↔	↔	↓	↗	↓	↗	↑	↗
sRANKL in the bone tissue at the distal femoral epiphysis	Cd1	↑		↑		↑		↑	
	Cd1 + AME	↑	↔	↑	↔	↔	↘	↔	↘
	Cd5	↑		↑		↑		↑	
	Cd5 + AME	↑	↔	↑	↔	↓	↘	↔	↘
sRANKL/OPG in the bone tissue at the distal femoral epiphysis	Cd1	↑		↑		↑		↑	
	Cd1 + AME	↑	↔	↔	↔	↔	↘	↓	↘
	Cd5	↑		↑		↑		↑	
	Cd5 + AME	↑	↔	↔	↘	↔	↘	↓	↘
Serum CTX	Cd1	↔		↔		↔		↑	
	Cd1 + AME	↔	↔	↔	↘	↔	↔	↑	↔
	Cd5	↔		↑		↑		↑	
	Cd5 + AME	↔	↔	↔	↘	↓	↘	↑	↘
**Bone biomechanical properties**									
Femoral neck – yield strength	Cd1	↔		↔		↔		↓	
	Cd1 + AME	↔	↔	↔	↔	↔	↔	↔	↗
	Cd5	↓		↔		↔		↓	
	Cd5 + AME	↔	↗	↔	↔	↔	↔	↑	↗
Femoral neck – fracture strength	Cd1	↔		↔		↔		↔	
	Cd1 + AME	↔	↔	↔	↔	↔	↔	↔	↔
	Cd5	↔		↔		↔		↔	
	Cd5 + AME	↔	↔	↔	↔	↔	↔	↑	↔
Femoral diaphysis – yield strength	Cd1	↔		↔		↓		↓	
	Cd1 + AME	↔	↔	↔	↔	↔	↗	↔	↗
	Cd5	↔		↔		↓		↓	
	Cd5 + AME	↔	↔	↔	↔	↔	↗	↔	↗
Femoral diaphysis – fracture strength	Cd1	↔		↔		↓		↔	
	Cd1 + AME	↔	↔	↔	↔	↔	↔	↑	↗
	Cd5	↔		↔		↓		↔	
	Cd5 + AM	↔	↔	↔	↔	↔	↔	↑	↗

[1] Detailed data on the impact of Cd and/or AME on the metabolism and bone biomechanical properties of female rats were published:

Brzóska, M.M.; Roszczenko, A.; Rogalska, J.; Gałażyn-Sidorczuk, M.; Mężyńska, M. Protective effect of chokeberry (*Aronia melanocarpa* L.) extract against cadmium impact on the biomechanical properties of the femur: a study in a rat model of low and moderate lifetime women exposure to this heavy metal. *Nutrients* **2017**, *9*, 543. https://doi.org/10.3390/nu9060543.

Brzóska, M.M.; Rogalska, J.; Gałażyn-Sidorczuk, M.; Jurczuk, M.; Roszczenko, A.; Tomczyk, M. Protective effect of *Aronia melanocarpa* polyphenols against cadmium-induced disorders in bone metabolism: A study in a rat model of lifetime human exposure to this heavy metal. *Chem. Biol. Interact.* **2015**, *229*, 132–146. https://doi.org/10.1016/j.cbi.2015.01.031.

ALP, alkaline phosphatase; BMD, bone mineral density; CTX, carboxy-terminal cross-linking telopeptides of type I collagen; OC, osteocalcin; OPG, osteoprotegerin; PC I, procollagen I; sRANKL, soluble receptor activator of nuclear factor-κB ligand; sRANKL/OPG, the ratio of sRANKL and OPG.

↑ – increase, ↓ – decrease, ↔ – no change compared to the control group

↗ – increase, ↘ – decrease, ↔ – no change compared to the respective group receiving Cd alone (Cd1 or Cd5)

Table S2. Exposure to cadmium (Cd) and/or supplementation with an extract from the berries of *Aronia melanocarpa* L. (AME) impact on the biomarkers of kidney damage in female rats. [1]

Parameters	Group	Experimental Duration							
		3 Months		10 Months		17 Months		24 Months	
Biomarkers of tubular damage									
KIM-1 in urine	Cd1	↑		↑		↑		↔	
	Cd1 + AME	↔	↘	↔	↘	↔	↘	↔	↘
	Cd5	↑		↑		↑		↑	
	Cd5 + AME	↔	↘	↔	↘	↔	↘	↔	↘
β2-MG in urine	Cd1	↔		↔		↑		↑	
	Cd1 + AME	↔	↔	↔	↔	↔	↘	↔	↘
	Cd5	↔		↔		↑		↑	
	Cd5 + AME	↔	↔	↔	↔	↔	↘	↔	↘
NAG in urine	Cd1	↔		↔		↑		↑	
	Cd1 + AME	↔	↔	↔	↔	↔	↘	↔	↘
	Cd5	↔		↑		↑		↑	
	Cd5 + AME	↔	↔	↔	↘	↔	↘	↔	↘
ALP in urine	Cd1	↔		↑		↑		↑	
	Cd1 + AME	↔	↔	↔	↔	↔	↔	↔	↔
	Cd5	↔		↑		↑		↑	
	Cd5 + AME	↔	↔	↔	↘	↔	↘	↔	↘
Biomarkers of glomerular damage									
ACR in urine	Cd1	↔		↔		↑		↑	
	Cd1 + AME	↔	↔	↔	↔	↑	↔	↔	↘
	Cd5	↔		↔		↑		↑	
	Cd5 + AME	↔	↔	↔	↘	↔	↘	↔	↘
PCR in urine	Cd1	↔		↑		↑		↑	
	Cd1 + AME	↔	↔	↔	↘	↔	↘	↔	↘
	Cd5	↔		↑		↑		↑	
	Cd5 + AME	↔	↔	↔	↘	↔	↘	↔	↘
Creatinine clearance	Cd1	↔		↔		↔		↔	
	Cd1 + AME	↔	↔	↔	↔	↔	↔	↔	↔
	Cd5	↔		↔		↓		↓	
	Cd5 + AME	↔	↔	↔	↔	↔	↗	↔	↗
Uric acid in the serum	Cd1	↑		↔		↔		↔	
	Cd1 + AME	↑	↔	↔	↔	↔	↔	↔	↔
	Cd5	↔		↔		↔		↑	
	Cd5 + AME	↔	↔	↔	↔	↔	↔	↔	↔
Urea in the serum	Cd1	↔		↔		↔		↔	
	Cd1 + AME	↔	↔	↔	↔	↔	↔	↔	↔
	Cd5	↔		↔		↑		↑	
	Cd5 + AME	↔	↔	↔	↔	↔	↔	↔	↘

[1] Detailed data on the impact of Cd and/or AME on the on the biomarkers of kidney damage in female rats were published: Smereczański, N.M.; Brzóska, M.M.; Rogalska, J.; Hutsch, T. The protective potential of *Aronia melanocarpa* L. berry extract against cadmium-induced kidney damage: A study in an animal model of human environmental exposure to this toxic element. *Int. J. Mol. Sci.* **2023**, *24*, 11647. https://doi.org/10.3390/ijms241411647.

ALP, alkaline phosphatase; ACR, albumin concentration in the urine adjusted for creatinine concentration; KIM-1, kidney injury molecule-1; NAG, *N*-acetyl-β-D-glucosaminidase; PCR, total protein concentration in the urine adjusted for creatinine concentration; β2-MG, β2-microglobulin.

↑ – increase, ↓ – decrease, ↔ – no change compared to the control group

↗ – increase, ↘ – decrease, ↔ – no change compared to the respective group receiving Cd alone (Cd1 or Cd5)

Table S3. Exposure to cadmium (Cd) and/or supplementation with an extract from the berries of *Aronia melanocarpa* L. (AME) impact on the concentration of calcium (Ca) in the serum and its urinary total excretion (TE–Ca) and fractional excretion (FE–Ca) in female rats.

Group	Experiment Duration			
	3 Months	10 Months	17 Months	24 Months
	Serum Ca (mg/L)			
Control	51.78<br>45.27–59.70	56.61<br>44.90–62.30	58.37<br>50.90–67.61	68.69<br>61.92–76.59
AME	47.57<br>42.13–61.56	61.52<br>43.99–74.65	50.58<br>37.06–57.78	67.99<br>61.81–75.15
Cd1	50.50<br>42.57–54.25	60.53<br>45.78–65.36	54.51<br>47.92–59.23	70.32<br>62.06–86.16
Cd1 + AME	45.32<br>34.54–58.33	65.76<br>49.24–80.72	62.06<br>46.45–65.98	70.11<br>54.66–72.75
Cd5	40.53 ^{a†}<br>36.24–48.03	60.95<br>49.70–68.30	55.90<br>49.42–66.90	67.34<br>61.32–74.64
Cd5 + AME	44.64<br>36.28–52.70	55.54<br>48.82–72.74	55.02<br>43.74–62.48	65.14<br>56.82–83.36
Effect size (η^2)	0.222			
	TE–Ca (mg/24 h)			
Control	0.906<br>0.406–1.764	2.226<br>0.700–2.833	3.375<br>0.193–5.937	3.867<br>2.400–6.166
AME	0.795<br>0.273–1.165	2.183<br>0.959–3.485	3.357<br>0.788–4.475	2.998<br>2.475–6.178
Cd1	1.304<br>0.224–3.466	1.071<br>0.389–2.106	2.491<br>0.526–3.534	5.546<br>3.252–7.104
Cd1 + AME	1.441<br>0.687–1.964	2.118<br>0.545–6.378	2.255<br>0.928–4.089	4.123<br>1.661– 6.035
Cd5	1.236<br>0.692–1.758	1.370<br>0.981–2.292	2.696<br>1.143–3.547	5.994 $^{a* b*}$<br>5.640–7.997
Cd5 + AME	1.699<br>0.477–1.981	2.130<br>0.634–4.886	2.322<br>1.768–3.362	4.634<br>2.366–6.209
Effect size (η^2)				0.186
	FE–Ca (%)			
Control	0.760<br>0.462–2.667	2.411<br>0.816–4.193	4.162<br>0.479–6.495	4.847<br>2.284–5.768
AME	0.901<br>0.549–1.667	2.423<br>0.722–4.292	4.712<br>1.844–7.316	4.498<br>1.818–6.508
Cd1	1.359<br>0.270–1.910	1.323<br>0.491–2.905	2.994<br>0.930–5.312	5.586<br>3.513–6.598
Cd1 + AME	1.991<br>0.753–4.150	2.093<br>0.475–9.909	2.720<br>0.906–6.348	3.741<br>1.954–5.309
Cd5	1.708<br>0.640–2.588	1.756<br>0.707–3.731	5.490<br>2.061–8.259	8.132 $^{a* b* d*}$<br>6.566–14.59
Cd5 + AME	1.717<br>0.554–3.232	2.381<br>0.695–6.389	3.174<br>2.018–5.795	2.984 ^{e†}<br>1.571–7.539
Effect size (η^2)				0.410

Data are shown as median and minimum and maximum values for eight animals (except for seven females in the AME, Cd 1, and Cd5 groups after 24 months). Statistically significant differences (Kruskal-Wallis test) compared to: a – Control group, b – AME group, d – Cd1 + AME group, and e – Cd5 group, where $^{*}p < 0.05$ and $^{\dagger}p < 0.01$, are marked.

Table S4. Exposure to cadmium (Cd) and/or supplementation with an extract from the berries of *Aronia melanocarpa* L. (AME) impact on the concentration of inorganic phosphorus (P_i) in the serum and its urinary total excretion (TE–P_i) and fractional excretion (FE–P_i) in female rats.

Group	Experiment Duration			
	**3 Months**	**10 Months**	**17 Months**	**24 Months**
	Serum P_i (mg/100 mL)			
Control	6.473<br>5.383–7.650	6.959<br>5.330–8.009	7.119<br>5.972–7.962	7.647<br>6.182–8.712
AME	6.313<br>5.493–7.345	6.275<br>5.105–7.990	7.526<br>5.907–8.416	7.687<br>7.423–8.566
Cd1	6.366<br>5.299–9.364	5.383<br>4.593–6.809	6.919<br>5.834–8.021	7.304<br>5.818–7.875
Cd1 + AME	6.068<br>5.213–11.55	6.902<br>4.766–9.727	7.651<br>6.695–7.700	7.216<br>5.969–8.316
Cd5	5.799<br>3.478–6.606	6.201<br>5.112–6.731	6.122 $^{a* b* d*}$<br>5.624–6.535	5.987 $^{a* b*}$<br>5.778–7.310
Cd5 + AME	4.962<br>3.368–6.159	6.208<br>4.627–7.778	7.473 ^{e*}<br>5.017–8.021	7.418<br>4.993–7.955
Effect size (η^2)			0.216	0.235
	TE–P_i (mg/24 h)			
Control	2.250<br>1.046–4.701	2.558<br>1.975–5.277	2.256<br>0.232–3.420	3.337<br>0.937–5.957
AME	2.014<br>0.923–3.675	2.820<br>1.982–3.175	3.107<br>0.527–4.760	2.855<br>0.609–5.706
Cd1	2.702<br>1.051–6.003	2.069<br>1.155–2.758	2.813<br>0.378–4.551	4.107<br>3.611–5.043
Cd1 + AME	2.394<br>0.987–5.515	2.033<br>0.467–2.988	2.505<br>1.029–5.383	3.761<br>1.086–7.738
Cd5	3.188<br>2.096–6.039	3.081<br>1.311–4.999	2.602<br>1.616–4.548	4.031<br>3.241–6.314
Cd5 + AME	2.080<br>1.171–2.856	3.575<br>1.271–7.606	1.421<br>0.506–2.836	3.449<br>1.234–6.871
Effect size (η^2)				
	FE–P_i (%)			
Control	1.550<br>0.754–4.206	2.474<br>1.947–2.944	2.324<br>0.448–3.554	3.842<br>0.607–6.313
AME	1.697<br>1.225–3.203	3.082<br>1.307–3.941	2.744<br>0.674–4.726	2.817<br>1.270–4.801
Cd1	1.982<br>1.381–2.835	2.340<br>1.787–3.955	2.125<br>0.697–5.660	4.240<br>3.629–6.193
Cd1 + AME	3.159<br>1.211–3.613	2.135<br>0.394–3.718	2.207<br>1.054–6.107	2.787<br>1.502–4.953
Cd5	3.038<br>1.566–8.333	3.342<br>1.334–7.912	5.176 ^{a*}<br>2.370–10.15	6.019 $^{a* b* d\dagger}$<br>5.220–17.73
Cd5 + AME	2.141<br>1.556–2.770	2.483<br>1.260–4.985	1.746 ^{e†}<br>0.460–3.463	2.956 ^{e†}<br>1.397–4.026
Effect size (η^2)			0.201	0.423

Data are shown as median and minimum and maximum values for eight animals (except for seven females in the AME, Cd 1, and Cd5 groups after 24 months). Statistically significant differences (Kruskal-Wallis test) compared to: a – Control group, b – AME group, d – Cd1 + AME group, and e – Cd5 group, where * $p < 0.05$, † $p < 0.01$, are marked.

Table S5. Exposure to cadmium (Cd) and/or supplementation with an extract from the berries of *Aronia melanocarpa* L. (AME) impact on the concentrations of 1,25-dihydroxyvitamin D_3 (1,25$(OH)_2D_3$), calcitonin (CT), and parathormone (PTH) in the serum of female rats.

Group	Experiment Duration			
	**3 Months**	**10 Months**	**17 Months**	**24 Months**
	1,25$(OH)_2D_3$ (nmol/L)			
Control	4.905	3.764	3.777	3.886
	3.241– 5.320	3.068–6.316	2.460–7.109	3.255–5.901
AME	4.136	2.955	3.711	4.213
	3.954–5.344	2.732–4.804	2.557–4.857	2.349–4.905
Cd1	4.632	2.445	2.771	3.166
	3.493–5.533	1.590–3.606	2.021–3.865	1.775–3.752
Cd1 + AME	3.533	2.587	4.952 ^{c*}	5.217 ^{c*}
	3.038–6.838	1.462–6.137	3.325–7.815	4.000–7.849
Cd5	3.154 ^{a*c*}	2.086 ^{a*}	2.535 ^{d*}	2.282 ^{d†}
	1.075–4.186	1.067–3.756	1.855–3.400	0.974–3.304
Cd5 + AME	4.163	3.486	4.263 ^{e†}	5.303 $^{c\dagger\ e\ddagger}$
	3.070–7.722	2.184–8.752	2.869–7.936	4.578–7.192
Effect size (η^2)	0.282	0.235	0.424	0.610
	CT (pg/mL)			
Control	128.1	101.5	161.0	96.48
	55.09–191.3	77.68–462.4	93.47–284.6	26.86–420.2
AME	138.4	98.15	121.2	90.63
	121.4–510.1	58.79–515.5	77.73–472.8	63.52–422.4
Cd1	109.2	121.4	49.17 $^{a\dagger\ b*}$	43.27
	95.59–161.7	87.43–522.5	9.666–97.73	23.58–73.08
Cd1 + AME	129.1	108.2	130.6 ^{c*}	100.3
	99.20–492.0	68.78–505.0	88.33–206.6	42.16–470.1
Cd5	99.96	117.0	60.61 $^{a\dagger\ d*}$	52.68
	69.20–144.4	65.80–532.2	42.01–92.25	27.86–101.1
Cd5 + AME	147.1	113.3	123.9 $^{c*\ e*}$	136.4 $^{c\dagger\ e*}$
	84.27–515.4	71.39–545.1	81.28–426.8	87.78–453.9
Effect size (η^2)			0.561	0.383
	PTH (pg/mL)			
Control	37.48	37.87	26.20	28.62
	28.41–55.56	15.25–59.50	4.312–62.78	14.82–42.62
AME	46.14	40.80	33.79	23.26
	38.33–52.11	17.11–137.1	16.43–60.72	20.11–57.05
Cd1	38.01	36.43	61.58 $^{a*\ b*}$	152.3 $^{a\dagger\ b\dagger}$
	28.84–251.3	20.33–154.0	51.82–176.4	125.4–189.8
Cd1 + AME	42.73	24.04	35.48 ^{c*}	42.35 ^{c*}
	31.35–99.10	14.55–159.9	23.55–49.48	13.80–52.87
Cd5	56.19	35.45	77.47 $^{a\dagger\ b*\ d\dagger}$	157.6 $^{a*\ b\dagger}$
	39.18–163.0	24.03–175.5	59.73–159.1	46.86–195.7
Cd5 + AME	48.64	50.00	45.22 ^{e*}	33.95 $^{c*\ e*}$
	29.65–68.27	36.80–192.6	28.65–54.64	22.10–63.94
Effect size (η^2)			0.538	0.595

Data are shown as median and minimum and maximum values for eight animals (except for seven females in the AME, Cd 1, and Cd5 groups after 24 months). Statistically significant differences (Kruskal-Wallis test) compared to: a – Control group, b – AME group, c – Cd1 group, d – Cd1 + AME group, and e – Cd5 group, where * $p < 0.05$, † $p < 0.01$, and ‡ $p < 0.001$, are marked.

Table S6. Exposure to cadmium (Cd) and/or supplementation with an extract from the berries of *Aronia melanocarpa* L. (AME) impact on the concentration of Klotho in the serum of female rats.

Group	Experiment Duration			
	**3 Months**	**10 Months**	**17 Months**	**24 Months**
	Klotho (pg/mL)			
Control	214.1<br>173.4–419.4	188.5<br>137.1–217.8	155.5<br>75.38–261.9	122.9<br>93.88–164.4
AME	176,4<br>163.3–205.7	178.1<br>149.2–231.9	156.1<br>47.50–284.4	116.7<br>103.7–184.9
Cd1	173.4<br>155.3–229.9	173.4<br>145.2–274.2	68.30<br>39.72–94.50	96.14<br>11.33–132.9
Cd1 + AME	166.3<br>119.0–225.8	184.5<br>167.3–237.9	197.8 ^{c*}<br>100.2–350.1	221.6 ^{c*}<br>96.14–410.0
Cd5	160.8 ^{a†}<br>141.1–175.4	188.5<br>151.2–225.8	42.33 $^{a† b† d‡}$<br>15.4–52.62	42.69 $^{a* d‡}$<br>11.33–92.61
Cd5 + AME	153.2 ^{a†}<br>141.1–201.6	228.8<br>173.4–266.1	125.4 ^{e†}<br>76.22–338.0	149.4 ^{e†}<br>75.38–535.2
Effect size (η^2)	0.331		0.577	0.459

Data are shown as median and minimum and maximum values for eight animals (except for seven females in the AME, Cd 1, and Cd5 groups after 24 months). Statistically significant differences (Kruskal-Wallis test) compared to: a – Control group, b – AME group, c – Cd1 group, d – Cd1 + AME group, and e – Cd5 group, where * $p < 0.05$, † $p < 0.01$, and ‡ $p < 0.001$, are marked.

Table S7. Exposure to cadmium (Cd) and/or supplementation with an extract from the berries of *Aronia melanocarpa* L. (AME) impact on the concentrations of 1,25-dihydroxyvitamin D_3 (1,25$(OH)_2D_3$) and 25-hydroxyvitamin D 1alpha-hydroxylase (1alpha-OHase) in the kidney of female rats.

Group	Experiment Duration			
	**3 Months**	**10 Months**	**17 Months**	**24 Months**
	1,25$(OH)_2D_3$ (nmol/g protein)			
Control	4.906<br>3.558–8.849	3.222<br>0.823–8.588	2.322<br>2.015–3.558	1.277<br>0.061–2.255
AME	4.532<br>3.414–9.012	3.833<br>2.954–5.092	1.543<br>1.222–3.728	1.352<br>1.168–2.489
Cd1	4.806<br>3.771–9.072	3.794<br>1.797–8.388	1.588<br>0.057–2.090	1.503<br>0.527–1.747
Cd1 + AME	5.363<br>2.925–8.858	2.674<br>1.748–3.743	1.358 ^{a†}<br>0.966–1.679	0.675 ^{b*}<br>0.152–0.842
Cd5	2.935 $^{a* c* d*}$<br>2.460–3.789	1.697 $^{a* b\dagger c\dagger}$<br>1.242–2.359	1.107<br>0.523–1.511	0.268 $^{a\dagger b\dagger c\dagger}$<br>0.039–0.700
Cd5 + AME	3.020 $^{a* b* c\dagger d*}$<br>1.834–3.802	2.043 ^{b*}<br>1.248–2.990	1.606 ^{a‡}<br>1.076–2.272	0.920<br>0.491–1.551
Effect size (η^2)	0.524	0.466	0.433	0.532
	1alpha-OHase (ng/mg protein)			
Control	0.441<br>0.287–0.815	0.320<br>0.224–1.049	0.464<br>0.337–0.653	0.389<br>0.271–0.706
AME	0.342<br>0.226–0.606	0.272<br>0.212–0.341	0.327<br>0.244–0.527	0.339<br>0.283–0.526
Cd1	0.433<br>0.244–0.808	0.366<br>0.186–0.719	0.336<br>0.242–0.421	0.270<br>0.190–0.731
Cd1 + AME	0.437<br>0.224–0.772	0.292<br>0.160–0.623	0.392<br>0.230–0.462	0.256<br>0.235–0.469
Cd5	0.212 ^{a*}<br>0.184–0.394	0.157 ^{a*}<br>0.115–0.428	0.295 ^{a*}<br>0.209–0.378	0.264 ^{a*}<br>0.171–0.341
Cd5 + AME	0.243 ^{a*}<br>0.179–0.332	0.175 ^{a*}<br>0.134–0.240	0.452 ^{e†}<br>0.398–0.546	0.455 ^{e†}<br>0.325–0.532
Effect size (η^2)	0.298	0.360	0.349	0.316

Data are shown as median and minimum and maximum values for eight animals (except for seven females in the AME, Cd 1, and Cd5 groups after 24 months). Statistically significant differences (Kruskal-Wallis test) compared to: a – Control group, b – AME group, c – Cd1 group, d – Cd1 + AME group, and e – Cd5 group, where * $p < 0.05$, $^{\dagger}p < 0.01$, and $^{\ddagger}p < 0.001$, are marked.
